# The midgut epithelium of mosquitoes adjusts cell proliferation and endoreplication to respond to physiological challenges

**DOI:** 10.1186/s12915-023-01769-x

**Published:** 2024-01-29

**Authors:** M. L. Taracena-Agarwal, B. Hixson, S. Nandakumar, A. P. Girard-Mejia, R. Y. Chen, L. Huot, N. Padilla, N. Buchon

**Affiliations:** 1 Department of Entomology, College of Agriculture and Life Sciences, Cornell Institute of Host-Microbe Interactions and Disease, Cornell University, Ithaca, NY 14852 USA; 2 Grupo de Biología y Control de Vectores, Centro de Estudios en Salud, Universidad del Valle de Guatemala, Guatemala City, 01015 Guatemala

**Keywords:** Midgut epithelium, Cell dynamics, DNA synthesis, Aedes, Anopheles, Culex, Polyploid cells, Endocycling, Endoreplication, Cell proliferation, Blood feeding

## Abstract

**Background:**

Hematophagous mosquitoes transmit many pathogens that cause human diseases. Pathogen acquisition and transmission occur when female mosquitoes blood feed to acquire nutrients for reproduction. The midgut epithelium of mosquitoes serves as the point of entry for transmissible viruses and parasites.

**Results:**

We studied midgut epithelial dynamics in five major mosquito vector species by quantifying PH3-positive cells (indicative of mitotic proliferation), the incorporation of nucleotide analogs (indicative of DNA synthesis accompanying proliferation and/or endoreplication), and the ploidy (by flow cytometry) of cell populations in the posterior midgut epithelium of adult females. Our results show that the epithelial dynamics of post-emergence maturation and of mature sugar-fed guts were similar in members of the *Aedes*, *Culex*, and *Anopheles* genera. In the first three days post-emergence, ~ 20% of cells in the posterior midgut region of interest incorporated nucleotide analogs, concurrent with both proliferative activity and a broad shift toward higher ploidy. In mature mosquitoes maintained on sugar, an average of 3.5% of cells in the posterior midgut region of interest incorporated nucleotide analogs from five to eight days post-emergence, with a consistent presence of mitotic cells indicating constant cell turnover. Oral bacterial infection triggered a sharp increase in mitosis and nucleotide analog incorporation, suggesting that the mosquito midgut undergoes accelerated cellular turnover in response to damage. Finally, blood feeding resulted in an increase in cell proliferation, but the nature and intensity of the response varied by mosquito species and by blood source (human, bovine, avian or artificial). In *An. gambiae*, enterocytes appeared to reenter the cell cycle to increase ploidy after consuming blood from all sources except avian.

**Conclusions:**

We saw that epithelial proliferation, differentiation, and endoreplication reshape the blood-fed gut to increase ploidy, possibly to facilitate increased metabolic activity. Our results highlight the plasticity of the midgut epithelium in mosquitoes’ physiological responses to distinct challenges.

**Graphical Abstract:**

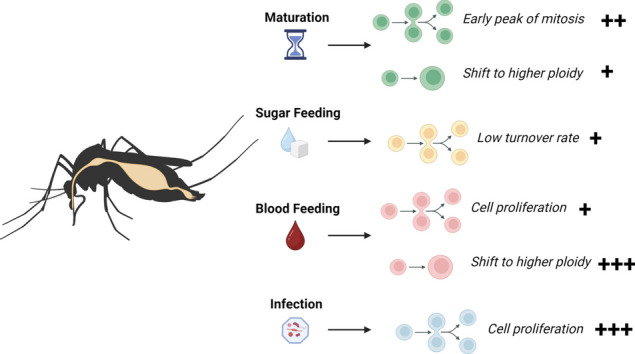

**Supplementary Information:**

The online version contains supplementary material available at 10.1186/s12915-023-01769-x.

## Summary

Mosquitoes transmit disease-causing pathogens during blood feeding, and the midgut epithelium is the first barrier viruses and parasites cross to complete their transmission cycle. Detailed studies of mosquitoes’ midgut cell composition and cell dynamics are scarce. We quantified mitotic events, endoreplication, and ploidy in the mosquito midgut epithelium in the post-emergence maturation period, as well as under conditions of sugar feeding, infection, and blood feeding. Our results indicate that the adult mosquito midgut possesses a population of progenitors, and that a basal level of cell proliferation is maintained in adulthood. Post-emergence midgut maturation is characterized by cell proliferation and an increase in enterocyte ploidy, resulting in a majority population of 16C enterocytes. Blood feeding increases epithelial proliferation in some species and endoreplication in others, in a blood-source dependent manner. The mosquito’s ability to regulate midgut cell dynamics could have direct implications for fitness and vector competence and could be relevant for disease-control.

## Background

The gut of a female hematophagous mosquito plays roles that are central to its survival, fitness, and vector competence. The gut epithelium not only digests the nutrients obtained from nectar or other sugar-based food sources, but also processes blood meals to fuel reproductive output. Crucially, as the organ that stores and digests the blood meal, the gut serves as the first interface between mosquito and pathogen and hence exerts influence over the vector’s potential to transmit disease. The gut epithelium is an active player in these, and other processes, making many alterations over the course of the mosquito’s lifecycle to support changing requirements.

The larval gut of a mosquito, which is of uniform width and adapted to an aqueous lifestyle and microbe-rich diet, gives rise to an adult gut with a narrow anterior and sac-like posterior midgut, respectively specialized in the digestion of nectar and blood [1, 2]. Immediately following blood meal ingestion, the epithelium engages in the rapid translation of early trypsins [3] as well as the transcriptional upregulation of peptidases to digest blood, peritrophin to assemble a peritrophic matrix, ferritins to sequester iron, as well as both immune effectors and modulators to achieve a fine-tuned response to the resulting proliferation of gut microbes [2, 4–6]. In response to various types of pathogenic challenge, the gut epithelium can not only activate local immune defenses [7], but can also recruit hemocytes to the basal lamina to generate a systemic immune response [8–10].

While developmental and transcriptional changes in the mosquito gut have been a subject of research for many years, one important dimension of gut physiology has only recently begun to receive attention: epithelial dynamics, a term which encompasses cell loss, proliferation, differentiation, and endoreplication in the gut epithelium. It has been well-established in the model insect *Drosophila melanogaster* that tissue-resident progenitors in the gut epithelium divide and differentiate in response to signals from their niche, downstream of changing nutritional inputs [11, 12], pathogenic challenges [13–15], and interactions with commensal gut microbes [15, 16]. These intestinal stem cells (ISCs) may either give rise to enteroblasts (EBs) which differentiate and endocycle to produce enterocytes (ECs) of various ploidies [17, 18], or they may differentiate into enteroendocrine progenitors (EEPs) which subsequently become enteroendocrine cells (EEs) [19]. Dividing ISCs can replace lost epithelial cells, and also plastically alter the size [11], composition [13], and ploidy profile [20] of the gut epithelium to adapt to changing conditions. Similar to *Drosophila,* the mosquito midgut epithelium comprises ECs, EEs, and a population of proliferative progenitors which, in *Aedes aegypti*, have been identified as putative ISCs/EBs [21]. However, much remains unknown about what role these progenitors play in host-microbe interactions, and how they may respond to the unique challenges posed by the hematophagous lifestyle.

Initially, midgut progenitor cells were thought to be mitotically inactive in adult mosquitoes [22], but in subsequent studies, mitoses have been observed in the adult midgut epithelia of *Anopheles*, *Aedes*, and *Culex* mosquitoes. Specifically, the proliferation of progenitor cells has been documented in newly emerged *Anopheles albimanus* [23], in *Anopheles stephensi* challenged with plasmodium [24], in *Ae. aegypti* challenged with oral bacterial pathogens and dengue virus (DENV) [25] and in *Aedes albopictus* and *Culex pipiens* after oral bacterial challenge or chemical damage [26, 27]. In the latter study, the authors did not find any mitotic cells in the midguts of *Anopheles gambiae* mosquitoes. However, the differences in methodology amongst these studies made the results difficult to compare to each other. To resolve this, in the present study, we developed a set of protocols that work not only under different physiological states, but also in different mosquito species.

Endoreplication, the process by which cells increase their DNA content without cell division, has also been documented in the midguts of several mosquito species. In a flow cytometry experiment, the *An. albimanus* midgut was shown to mature from mostly diploid (2C) to predominantly tetraploid (4C) and octaploid (8C) in the time between 6- and 12-h post-emergence (PE) [23]. Putative endoreplication has also been observed in the *An. albimanus* midgut epithelium in response to systemic inoculation with *Serratia marcescens* [28] and oral challenge with *Plasmodium berghei* [29]. Likewise, oral DENV-2 infection in *An. albimanus* and *Ae. aegypti* stimulated DNA synthesis without mitosis in the midgut epithelium of both mosquitoes [30]. Altogether, these examples add evidence to suggest that the mosquito midgut could effect changes in epithelial ploidy in response to various types of challenge. However, very few studies have included more than one or two conditions in the same species to establish baselines and to identify the nature of these responses in a wider context.

Hematophagy is of special importance in both the lifecycle of mosquito vectors and the transmission cycle of vector-borne diseases, and some reports support a role for epithelial dynamics in the midgut’s response to blood feeding. Okuda et al. documented apoptotic cell elimination in the midgut of *Culex quinquefaciatus* in response to blood feeding and observed differentiation of basal regenerative cells by electron microscopy. Using cell counts measured by optical microscopy, they reported a gradual reduction of the regenerative cell population over the course of three to four blood meals [31]. From these observations it was concluded that a finite pool of progenitor cells can be activated to differentiate and replace cells lost during the digestion of blood. Quantification of mitotic cells by phosphorylated Histone-3 (PH3) staining in blood-fed *Ae. aegypti* mosquitoes has been used to show the protective role of the peritrophic matrix during blood meal digestion. Blocking the formation of the peritrophic matrix resulted in a significant increase in the numbers of mitotic cells when bacteria were present [25]. The authors reported that while the number of mitotic cells during the time of the digestion of blood is low, the progenitor cells present in the posterior midgut epithelium could still be stimulated to divide if chemical stress was promoted by addition of paraquat to the blood or if the sequestration of the blood bolus from the epithelium was compromised. In contrast, Cui and Franz, working with the Higg’s White Eye strain of *Ae. aegypti*, found, using single cell sequencing, that the proportion of progenitor cells in the midgut was significantly increased at 24 h post-blood meal (PBM). This likely indicates a proliferative response to the blood meal, even without disruption of the peritrophic matrix [21]. In the *Ae. albopictus* midgut, blood feeding induced the phosphorylation of extracellular signal-regulated kinase (ERK), an Epidermal growth factor receptor (EGFR) pathway kinase [32] which, when it occurs in Drosophila ISCs, is sufficient to drive proliferation [33]. We and others have previously argued that epithelial dynamics in the mosquito midgut may have profound effects on mosquitoes’ fitness as well as their ability to transmit disease: Epithelial cell loss may help to bottleneck invading plasmodium parasites [34, 35], and viral pathogens [36–39], while endoreplication in ECs may enhance the gut’s capacity to transcribe immune effectors by increasing copy number [28–30]. The proliferation and differentiation of new epithelial cells to replace cells lost due to pathogenic exposure may help mosquitoes tolerate the effects of oral infection, prolonging their survival and, thereby, increasing their vectorial capacity [27, 40]. Finally, the gut could adapt its epithelial composition in response to nutritional or hormonal cues to optimize the digestion and absorption of blood meals, with ramifications for fecundity and fitness [40].

In this study, we sought to comprehensively describe and directly compare cellular dynamics in the mosquito midgut epithelium of major vector species of the *Aedes*, *Culex*, and *Anopheles* genera in the contexts of post-emergence maturation, oral bacterial infection, and blood feeding. For this purpose, we used PH3 immunostaining to label mitotic cells, allowing us to quantify the amount of cell proliferation in process at any given time. We also employed the incorporation of nucleotide analogues to visualize cells that contain newly synthesized DNA, allowing us to trace new epithelial cells that arise as progeny of stem cells, and to detect ECs that have undergone endoreplication. Additionally, we used flow cytometry to analyze the cell populations in the midgut by quantifying DNA content, allowing us to profile the ploidy of epithelial cells, and measure ploidy changes in response to physiological challenge. Our results show that mosquitoes use condition-specific dynamics that involve proliferation and endoreplication, producing changes in the cellular composition of the midgut epithelium as a response to infection and blood feeding.

## Results

### The mosquito midgut epithelium is a tissue with active cell proliferation

**Fig. 1:**
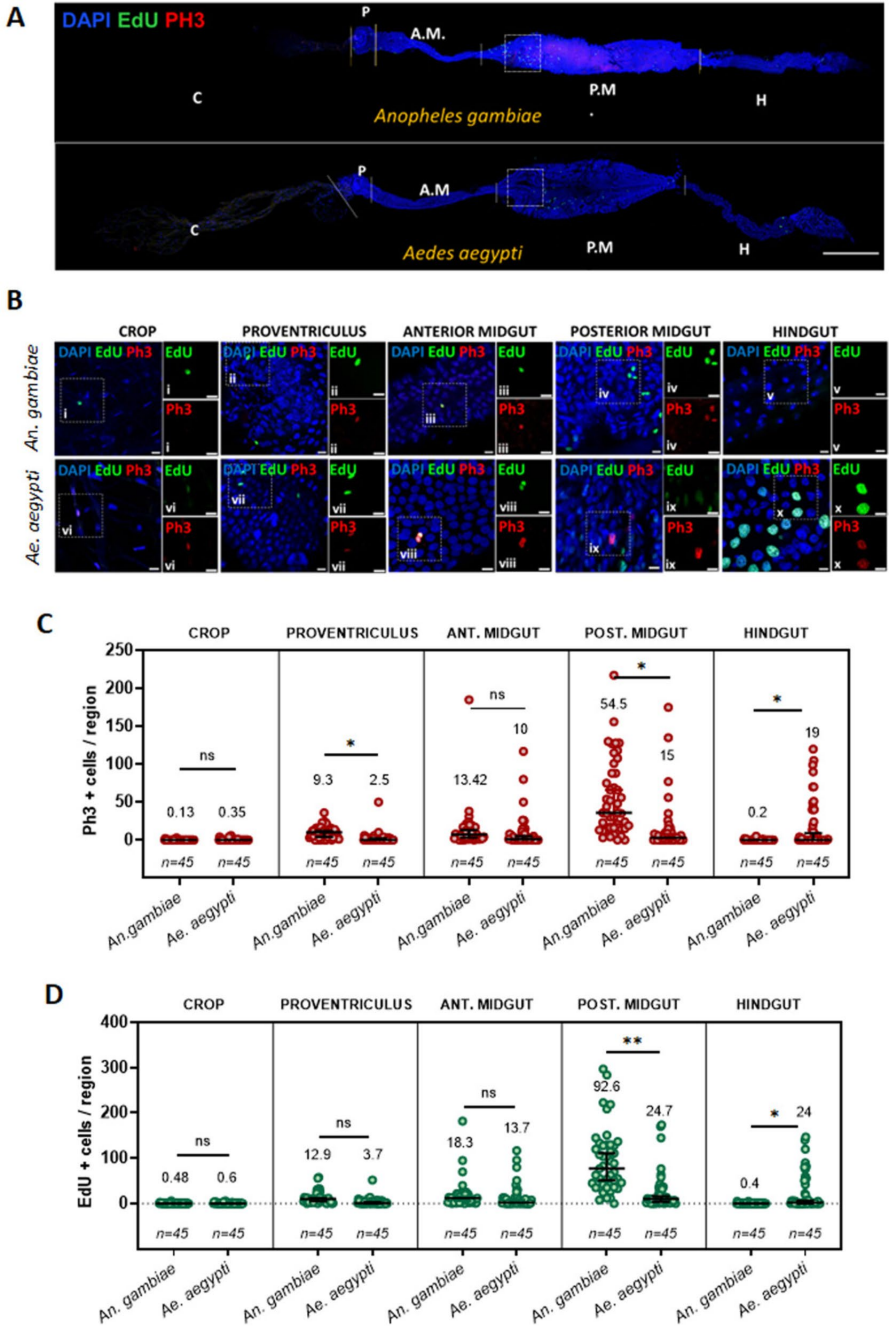
Cell turnover occurs in the gut epithelium of sugar-fed *Anopheles gambiae* and *Aedes aegypti* mosquitoes. Five-day-old females were maintained for 72 h on a diet of 10% sucrose supplemented with EdU prior to dissection. Guts were treated with a Click-iT cocktail to label EdU (green) and stained with an anti-PH3 antibody (red) and DAPI (blue). Cell counts of EdU-positive and PH3-positive cells were obtained for crop, proventriculus (Pv.), midgut (anterior and posterior) and hindgut. Representative images for the whole gut of *An. gambiae* and *Ae. aegypti* are shown in (**A**), (scale bar = 500 µm), with a dotted box designating the region of interest used in this study to quantify EdU incorporation (Figs. 2, 3, 4, 5 and 6). Magnified images of each region are shown in (**B**), (scale bar = 10 µm). Total counts of PH3-positive (**C**) and EdU-positive cells (**D**) were quantified in each gut region. Results are from at least three biological replicates. Values on top indicate mean values, and error bars are SEM. Three biological replicates were made, and graphs show all replicates combined. Statistics: Mann–Whitney test; *, **, and *** respectively indicate *P* values of < 0.05, < 0.001, and < 0.001

To establish the baseline levels of epithelial turnover in the adult female gut, we used two complementary approaches to quantify epithelial dynamics. First, immunostaining against phospho-histone H3 (PH3) is a direct indicator of mitosis and therefore a proxy for the rate of ISC proliferation; second, the nucleotide analog 5-Ethynyl-2′-deoxyuridine (EdU), which may be supplied in the diet, acts as a cumulative marker that is integrated in all new cells as well as maturing ECs that undergo endocycling [41]. We fed five-day-old mosquitoes with a sugar solution containing EdU for 3 days and quantified PH3-positive and EdU-positive cells in the crop, proventriculus, anterior and posterior midgut, and hindgut of *Ae. aegypti* (Liverpool (Lvp) strain) and *An. gambiae* (*s.l.,* G3 strain) (Fig. 1). We detected PH3- and EdU-positive cells in most of the compartments mentioned (Fig. 1C-D) with the exception of the crop and the *An. gambiae* hindgut, which were almost completely quiescent. Since each region is differently sized, and comprises a different number of cells, we normalized the net counts to the total cell number per region (Additional file 1: Fig. S1). This analysis revealed that the proventriculus of *An. gambiae* presented basal levels of PH3- and EdU-positive cells approximately two times higher than any other region in the gut. In *Ae. aegypti*, the hindgut displayed the highest percentage of PH3- and EdU-positive cells. However, the PH3-positive nuclei detected in this region were too large to be diploid, were not basally located, and lacked the condensed chromatin states characteristic of the mitotic phases with known presence of PH3 (prophase, metaphase, anaphase, or early telophase), and therefore did not present the appearance of mitotic progenitors (Fig. 1B). Given these characteristics, it is likely that the phosphorylation of the Histone H3 in these cells is related not to cell division but rather to transcriptional regulation [42, 43].

**Fig. 2:**
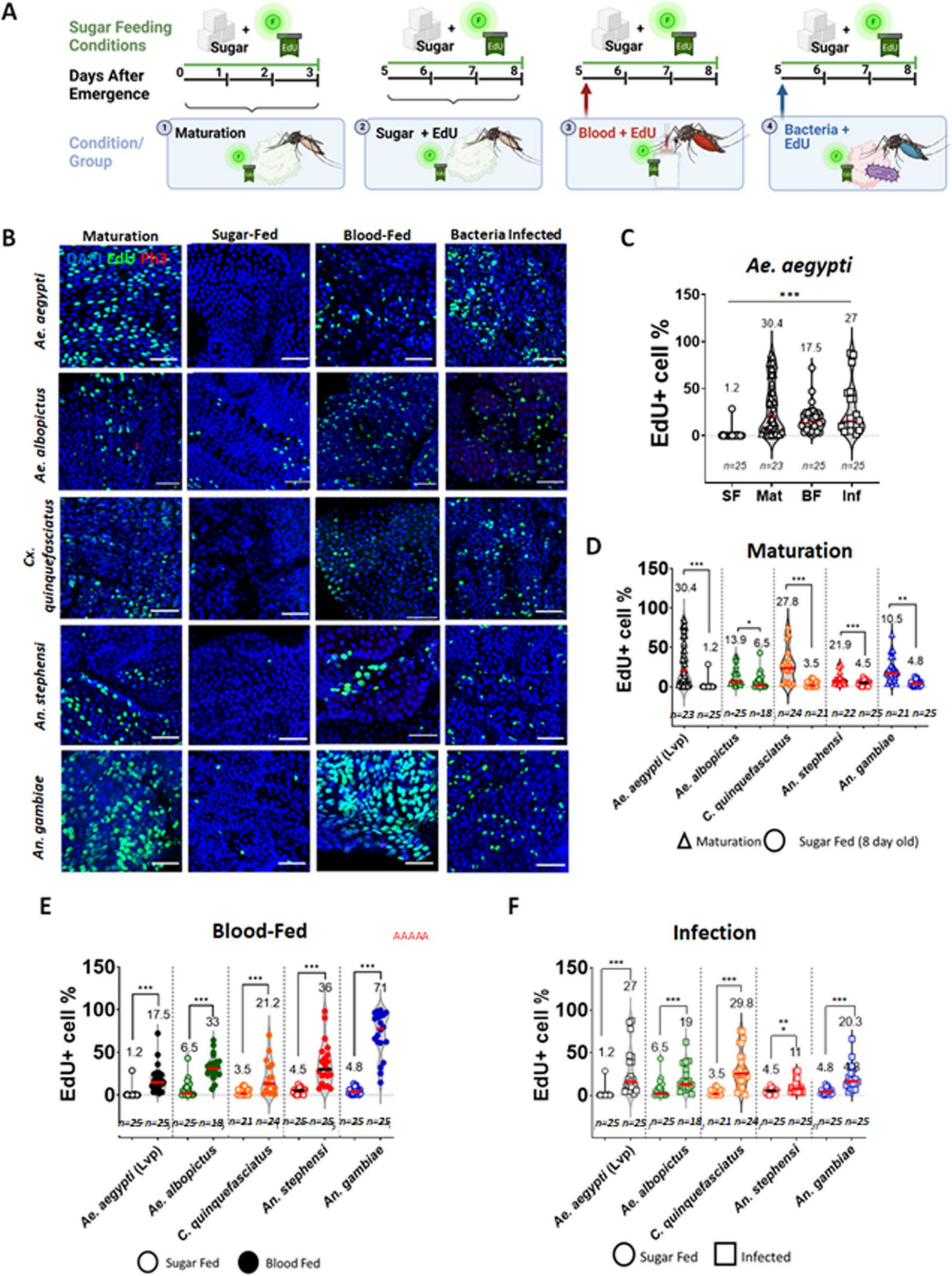
The midgut epithelium of adult female mosquitoes shows conserved post-emergence maturation, and dynamic adjustment to changing physiological conditions. Experimental design: **A** Emerging mosquitoes received EdU in pupal water (maturation experiment). Five-day old mosquitos received EdU in a blood meal (blood feeding experiment), a sucrose-baited suspension of *Pseudomonas entomophila* (infection experiment) or neither (sugar feeding baseline experiment). All were maintained on 10% sucrose supplemented with EdU for 72 h prior to dissection. Guts were treated with a Click-iT cocktail to label EdU (green) and stained with an anti-PH3 antibody (red) and DAPI (blue). Representative images of the posterior midgut region of interest (ROI) for each condition in all mosquito species are shown in (**B**) (scale bar = 50 µm). To illustrate the amplitude of responses to different stimuli in a single mosquito species, the percentages of EdU-positive cells in the posterior midgut ROI in *Aedes aegypti* across all conditions are shown in (**C**). Values on top indicate mean values and error bars are SEM. Statistics: one-way ANOVA, *P* < 0.001. A full graph containing all comparations by species is shown in Fig. S5. Percentages of EdU-positive cells in the ROI, relative to the percentages in the sugar-fed condition are shown for maturing (**D**), blood-fed (**E**), and infected (**F**) females of all species. Results are from at least three biological replicates. Values on top indicate mean values, and error bars are SEM. Statistics: Mann–Whitney test; *, **, and *** respectively indicate *P* values of < 0.05, < 0.001, and < 0.001

**Fig. 3:**
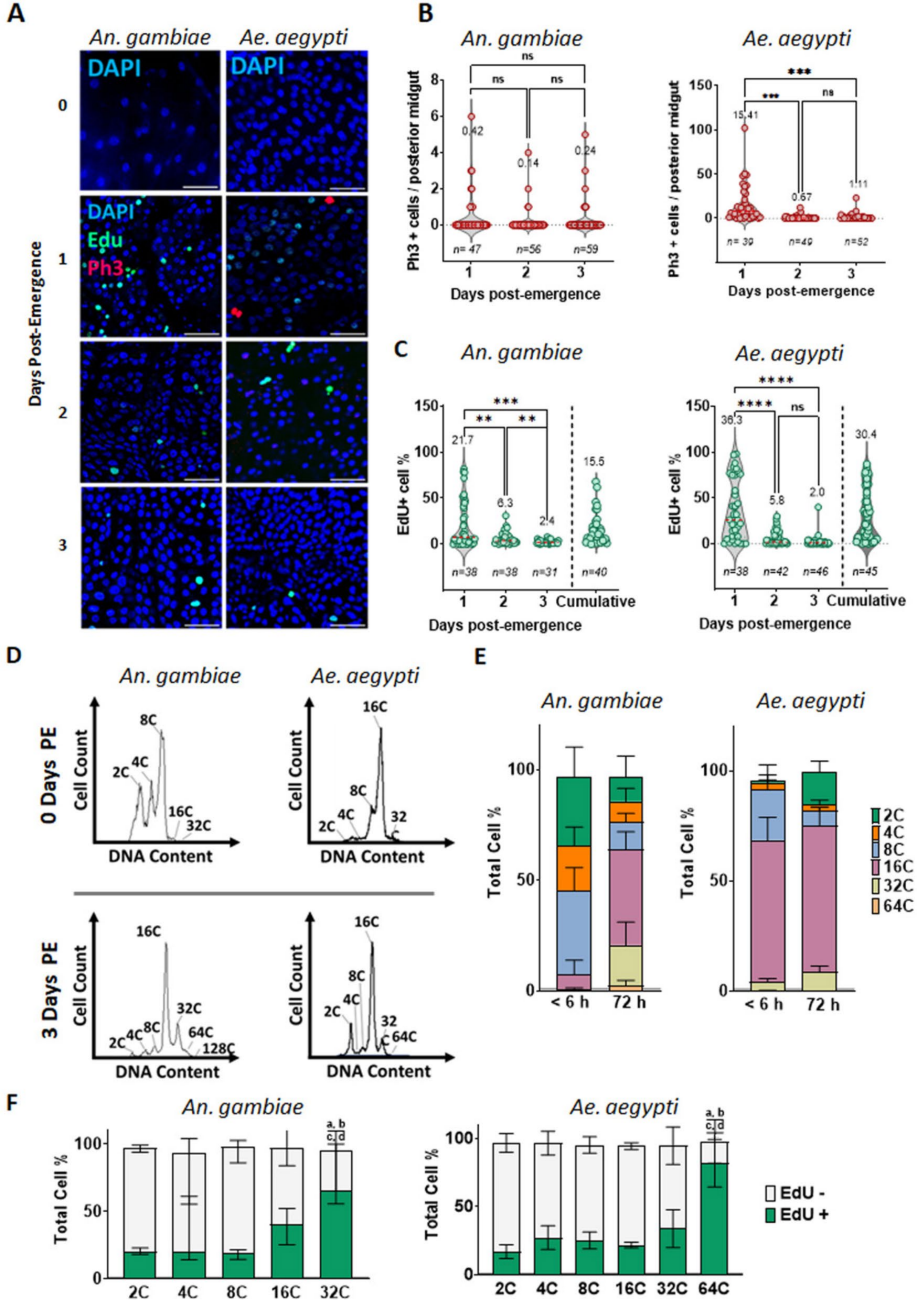
The midgut of a newly emerged female mosquito undergoes a rapid species-specific maturation process characterized by epithelial proliferation and/or endoreplication. The guts of 1, 2, and 3-day old sucrose-fed *Aedes aegypti* and *Anopheles gambiae* were dissected immediately following 24 h on a diet supplemented with EdU (administered, respectively, from pupal water to 24 h, 24–48 h, and 48–72 h), and are depicted after Click-iT EdU-labeling (green), anti-PH3 (red) and DAPI (blue) staining. Day 0 guts were dissected within six hours of emergence and stained with DAPI only (**A**) (scale bar = 50 µm). **B** Quantification of PH3-positive cells revealed higher levels of mitosis at 24 h post-emergence in *Ae. aegypti* but not *An. gambiae*. **C** In both species, EdU incorporation primarily occurred over the first 24 h post emergence. Plots depict the percentage of EdU-positive cells accumulated in the posterior midgut region of interest over 24 h of exposure in 1-, 2-, and 3-day-old mosquitoes, alongside percentages from mosquitoes which received EdU continuously (initially in pupal water, subsequently in sucrose) for 72 h post-emergence (cumulative). Results are three biological replicates. Values on top indicate mean values, and error bars are SEM. Brown-Forsythe and Welch ANOVA (analysis of variance) test for multiple comparisons was utilized. Dunnett’s T3 multiple comparisons test for multiple comparisons; *, **, and *** respectively indicate *P* values of < 0.05, < 0.001, and < 0.001. **D** Flow cytometry data from mosquitoes continuously exposed to EdU from the pupal phase showed that the cell populations of dissected posterior midguts at day 3 were significantly altered compared to day 0 in both *An. gambiae* and *Ae. aegypti.* Representative histograms are shown. Cell count in the Y-axis is normalized to the total number of cells. Ploidy is indicated at the top of each peak, and gating strategy with diploid controls can be found in Fig. S2. **E** Cell percentage relative to each peak of DNA content was plotted as stacked bar plots, equalizing values to a 100%. **F** In each cell population, percentages of EdU-positive cells were plotted (median with 95% CI) in stacked bars. Percentages of EdU-positive cells, from each cell population based on nuclei size, show that the cell populations ≥ 32C had the largest percentages of EdU incorporation. For flow cytometry, samples consisted of pools of 40 posterior midguts at day 0 and 25 posterior midguts at day three, *n* = 9 samples per condition, from at least three biological replicates. Brown-Forsythe and Welch ANOVA (analysis of variance) test for multiple comparisons was utilized to compare EdU positive percentages between ploidy groups. Groups were assigned ID as follows: 2C = a, 4C = b, 8C = c, 16C = d, 32C = e. Letters at the top of each bar indicate which groups are significantly different using Dunnett’s T3 multiple comparisons test for multiple comparisons

**Fig. 4:**
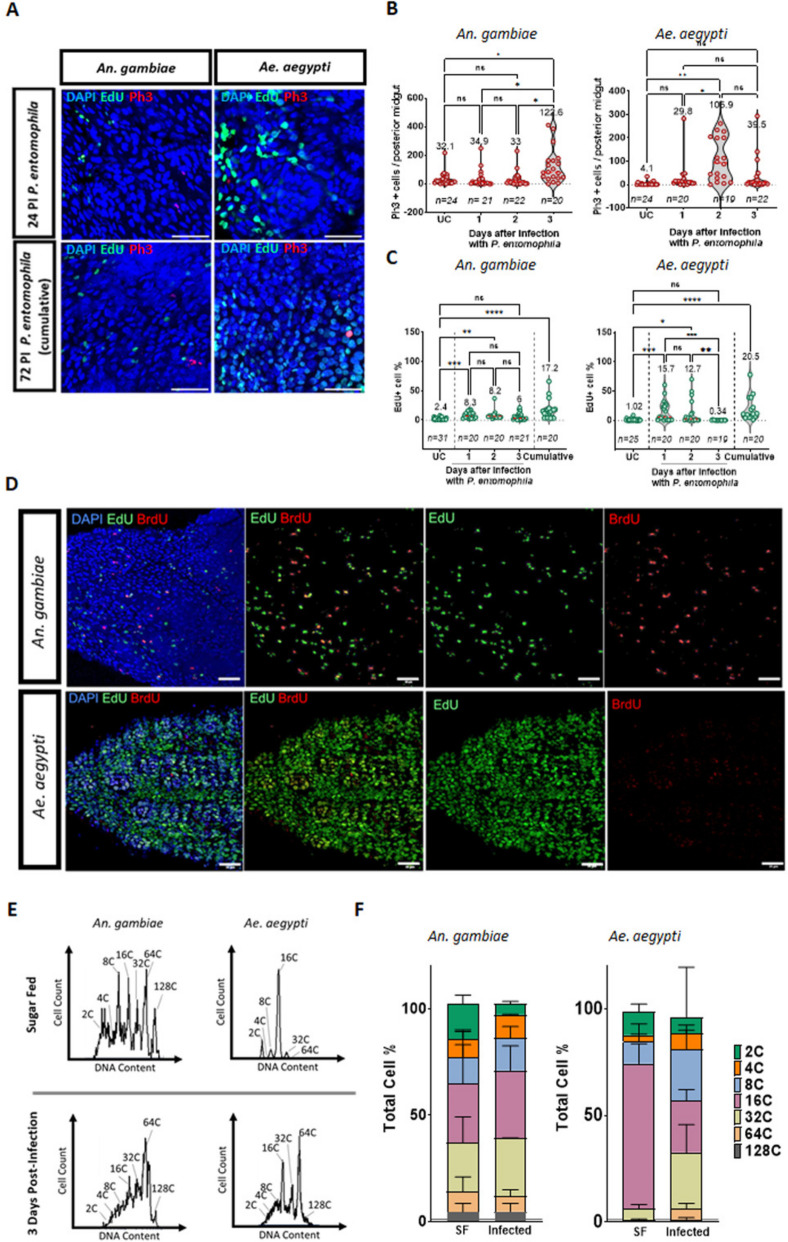
Oral infection with *Pseudomonas entomophila* induces mitosis and ploidy changes in *Anopheles gambiae* and *Aedes aegypti*. Mature, 5-day-old females were infected with a sucrose-baited solution containing *P. entomophila* (OD_600_ 100) and EdU, thereafter maintained on sucrose/EdU and dissected at 24- or 72-h post-infection (PI). Guts were treated with a Click-iT cocktail to label EdU (green) and stained with an anti-PH3 antibody (red) and DAPI (blue). Representative images of *An. gambiae* and *Ae. aegypti* at each timepoint are shown (**A**) (scale bar = 50 µm). For PH3 quantifications (**B**) and 24-h EdU pulse experiments (**C**) midguts were dissected from mosquitoes that were maintained on EdU from 0–24 h (day 1), 24–48 h (day 2) or 48–72 h (day 3) PI. EdU-positive cells were quantified in the posterior midgut region of interest (ROI). Cumulative EdU incorporation in the ROI in unchallenged (UC) mosquitoes and mosquitoes orally infected with *P. entomophila*/sucrose/EdU and maintained on sucrose/EdU over the full 72 h PI was also quantified. Results are from at least three biological replicates. Values on top indicate mean values. Statistics: Mann–Whitney test; *, **, and *** respectively indicate *P* values of < 0.05, < 0.001, and < 0.001. *Ae. aegypti* accumulated the greatest number of EdU-positive cells in the first day, with the rate of incorporation falling on the third day, while the rate of incorporation remained steady in *An. gambiae* across all three days. This was confirmed also by an EdU/BrdU switch assay, where after the first 24 h PI, mosquitoes were switched from EdU (green) to BrdU (red) (**D**). Most of the cells in the *Ae. aegypti* posterior midgut incorporated EdU, confirming that there was a period of intensive DNA synthesis during the first 24 h PI. In *An. gambiae,* the incorporation of EdU and BrdU was comparable, confirming a more modest but sustained response. Flow cytometry data showed that the cell populations from non-infected mosquitoes (sugar-fed) and infected mosquitoes were significantly different from each other*.* Representative histograms are shown in (**E**). Cell count in the Y-axis is normalized to the total number of cells. Ploidy is indicated at the top of each peak. Relative percentages of the total number of events were graphed as stacked bar plots to present the portion of each population relative to the total (**F**). Samples consisted of pools of 8 posterior midguts, *n* = 9 samples per condition, from at least three biological replicates

**Fig. 5:**
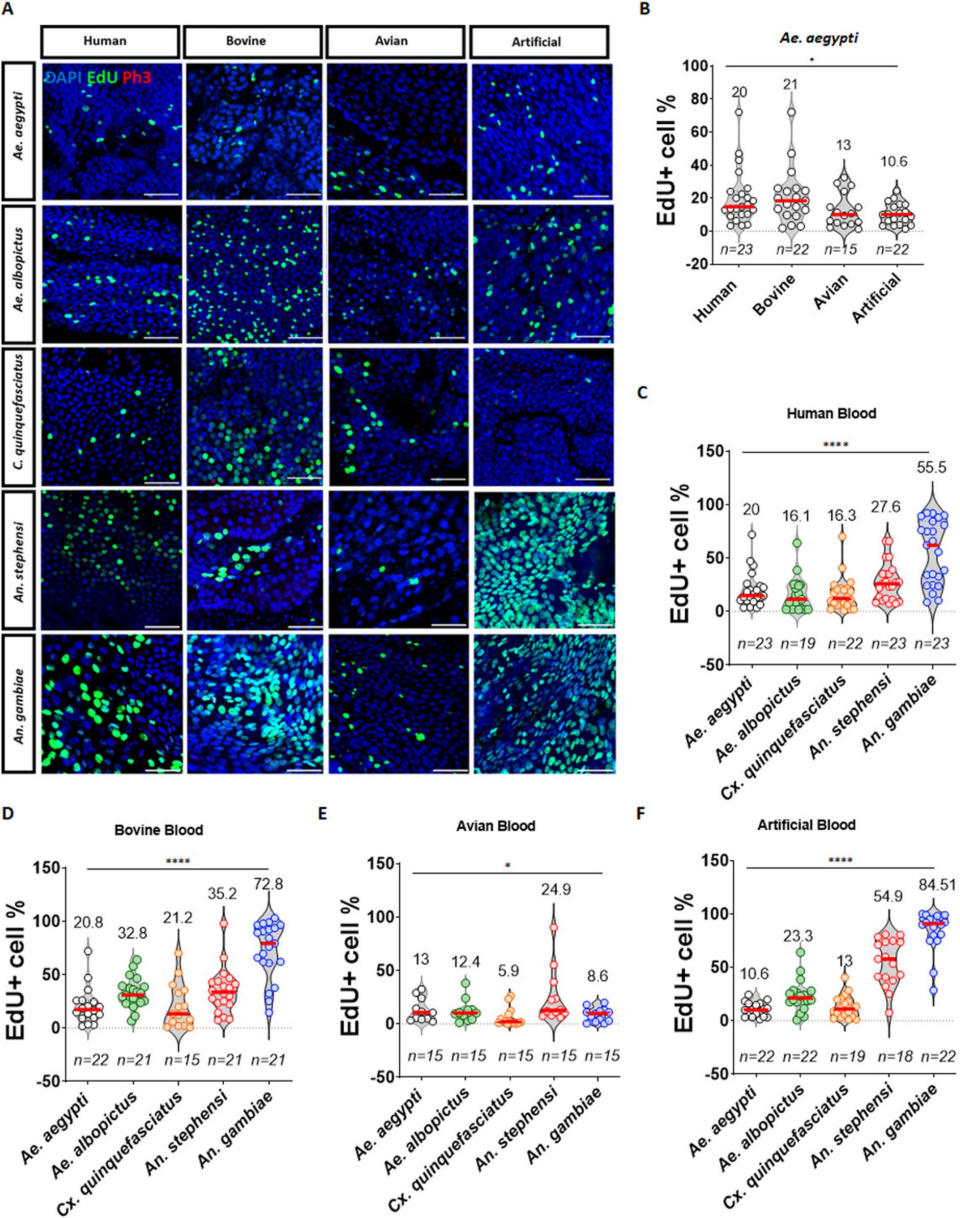
The mosquito response to the blood meal is source-dependent. Five-day old mosquitoes were blood fed on different sources of blood: human, bovine *(Bos taurus),* avian *(Gallus gallus)* and artificial (Skito-Snack). Blood meals were supplemented with EdU, and blood-fed mosquitoes were maintained on sucrose/EdU for 72 h prior to dissection. Guts were treated with a Click-iT cocktail to label EdU (green) and stained with an anti-PH3 antibody (red) and DAPI (blue). Representative images of the posterior midgut region of interest (ROI) at 72 h post-blood feeding are shown in (**A**), scale = 50 µm. Quantification of the percentage of EdU-positive cells in the ROI in *Aedes aegypti* after feeding with the four different blood sources (**B**) showed that human and bovine blood induced stronger responses in this mosquito when compared with avian blood or the artificial blood meal. When comparing the responses of different species to the same blood-source (**C-F**), *Anopheles gambiae* showed the strongest response to all but avian blood (**E**). Results are from at least three biological replicates. Values on top indicate mean values, and error bars are SEM. Statistics: Mann–Whitney test; *, **, and *** respectively indicate *P* values of < 0.05, < 0.001, and < 0.001

**Fig. 6:**
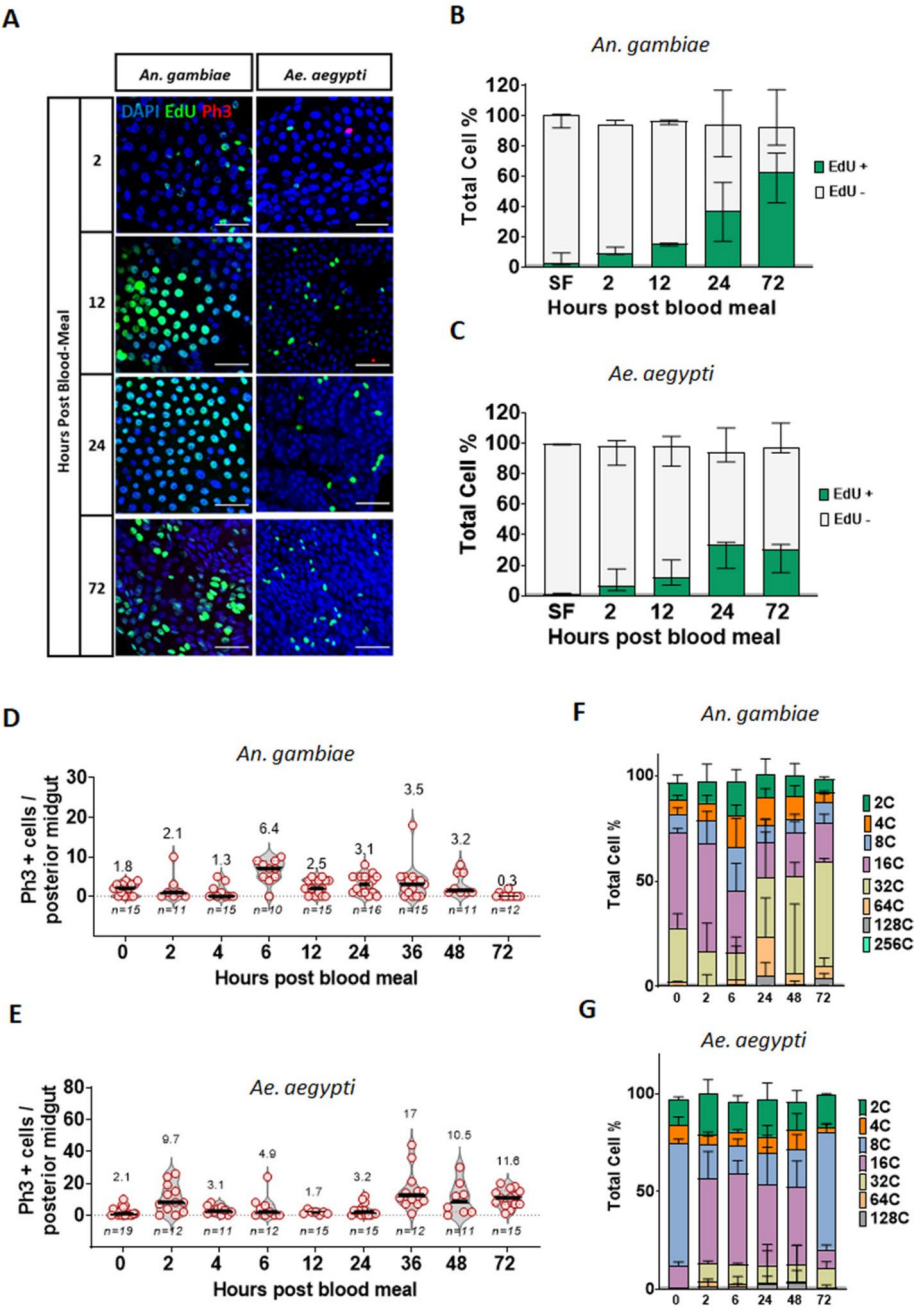
Human blood triggers rapid DNA synthesis in the midgut epithelium of *Anopheles gambiae* and *Aedes aegypti* leading to increases in ploidy. Adult mosquitoes were fed on human blood supplemented with EdU and continuously maintained on a diet of sucrose/EdU for up to 72 h prior to dissection. Guts were treated with a Click-iT cocktail to label EdU (green) and stained with an anti-PH3 antibody (red) and DAPI (blue). Representative images of the posterior midgut region of interest (ROI) at 2, 12, 24 and 72 h post-blood meal (PBM) are shown (**A**), scale bar = 50 µm. **B**-**C** Quantification of EdU-positive cells using flow cytometry at 2,12, 24 and 72 h post-blood meal also shows early incorporation of EdU after the blood meal in *An. gambiae* and *Ae. aegypti.* **D**-**E** Quantification of PH3-positive cells in the posterior midgut after 2, 4, 6, 12, 24, 36, 48 and 72 h did not reveal any timepoint at which proliferation was high enough to account for the DNA synthesis observed in *An. gambiae*. Results are from at least three biological replicates. Values on top indicate mean values, and error bars are SEM. **F** Flow cytometry analysis shows that upon blood feeding, larger ploidy cells (> 32C) were generated in *An. gambiae*, effecting a persistent change to epithelial structure. **G** *Ae. aegypti* mosquitoes also showed a significant increase of 16C and > 32C cells, but this effect appeared to be transient as the cell population at 72 h post-blood meal reverted to the ploidy profile observed in the sugar-fed epithelium, with the larger cells generated during the peak of the digestive process appearing to be lost

The posterior midgut is of particular interest in mosquito vectors, as it serves as the primary site for blood storage and digestion and as the entry point for most vectored pathogens. The average number of PH3-positive cells per posterior midgut was 54.5 (SEM = 7.5) in *An. gambiae,* and 15 (SEM = 5.1) in *Ae. aegypti* (Fig. 1C)*.* PH3 counts were below 1% of the total cell number in both species, and EdU counts were below 5% of cells overall. Interestingly, the percentage of EdU-positive cells in *An. gambiae* was at least seven times higher than in *Ae. aegypti* (Additional File 1: Fig. S1C)*.* In both species’ posterior midguts, the highest concentration of EdU incorporation was observed proximal to the anterior/posterior midgut boundary. We therefore designated this portion of the posterior midgut as the region of interest (ROI) for EdU quantifications in our study (Fig. 1A). All EdU-positive cell percentages measured by microscopy in the remainder of this study (Figs. 2, 3, 4, 5 and 6) reflect the percent within this designated ROI. All PH3 counts reflect the number of PH3-positive cells in the whole posterior midgut. Overall, our results indicate that epithelial dynamics and mitotic activity occur in the midgut of adult female mosquitoes at low rates, with a basal rate of new cell accumulation that ranges from 1–5% of the total number of cells in a three-day period, with rates varying between the regions of the gut.

### *Aedes, Anopheles* and *Culex* adult midguts actively regulate epithelial dynamics in reaction to blood feeding and infection

In the Drosophila midgut epithelium, stem cell proliferation is regulated to replenish damaged cells and preserve epithelial integrity [16, 44]. In addition, it has been documented that challenges like oral infection with pathogenic bacteria result in the appearance of ECs of higher ploidy [20], suggesting that cells built by ISCs can regulate their ultimate ploidy based on physiological conditions. It remains to be fully described whether, and under what circumstances, epithelial dynamics in the mosquito midgut could be similarly regulated. To comprehensively characterize epithelial dynamics in the midguts of hematophagous mosquito vectors, we selected five different species of medically relevant mosquitoes (*Ae. aegypti, Ae. albopictus, Cx. quinquefasciatus, An. gambiae* and *An. stephensi*), and quantified proliferation, EdU incorporation (in the ROI), and changes in ploidy in the posterior midgut epithelium (a) in the immediate aftermath of emergence (b) during maintenance on a diet of sugar (c) after oral infection with entomopathogenic bacteria and (d) during blood meal digestion (Fig. 2).

To determine if epithelial changes occurred in the adult gut immediately post-emergence, we fed newly-emerged mosquitoes with sugar supplemented with EdU for three days (Fig. 2A) and compared the results to eight-day-old mosquitoes fed on sugar with EdU for the same amount of time (Fig. 2C-D). We found that all the mosquito species studied presented significantly higher numbers of EdU-positive cells in the first days after emergence, suggesting that the mosquito posterior midgut epithelium goes through an active period of proliferation and differentiation once it has emerged, but that the rate of EdU integration is more modest in mature guts. The intensity of the post-emergence EdU integration was highest in *Ae. aegypti* and *Cx. quinquefasciatus* mosquitoes involving 30.4% (SEM = 4.2) and 27.8% (SEM = 5.3) of cells in the ROI, respectively. Consistent with the results obtained in the quantification of EdU-positive cells in *Ae. aegypti* and *An. gambiae* (Fig. 1D), mature *Ae. albopictus, Cx. quinquefasciatus* and *An. stephensi* mosquitoes that were fed on sugar alone showed low levels of EdU-positive cells in the ROI (6.33% (SEM = 2.1), 3.5% (SEM = 0.82) and 4.8% (SEM = 0.64) respectively). Quantification of PH3-positive cells in sugar-fed conditions was also modest (Additional file 2: Fig. S2). Overall, these low levels of PH3- and EdU-positive cells suggest a low level of basal turnover in all species.

The low rate of turnover in the posterior midguts of sugar-fed mosquitoes contrasted sharply with the responses to oral bacterial infection. To study the response to oral infection, a concentrated solution of *P. entomophila* (OD_600_ 100) baited with sugar and supplemented with EdU was supplied for a period of 4 h, after which the mosquitoes were maintained on sugar with EdU alone (Fig. 2A). An increase of PH3 (Fig. S4A and C) and EdU accumulation (Fig. 2F and Additional file 3: Fig. S3) was observed 72 h after the bacterial challenge in all the mosquito species studied. Means of PH3 counts increased at least two-fold when quantified 3 days after the infection event compared to an uninfected baseline (*Ae. aegypti P* = 0.0667, *Ae. albopictus P* = 0.0884, *Cx. quinquefasciatus P* = 0.0224, *An. stephensi P* = 0.1451, *An. gambiae P* = 0.0046, Welch’s t test, where *P* < 0.05 is significantly different). Taken together, the PH3-positive and EdU-positive cell counts suggested that the kinetics of the epithelial response vary significantly between species: The number of EdU-positive cells in *Ae. aegypti* at 72 h PI was about twenty times higher than the basal count under sugar-fed conditions, suggesting a very fast turnover rate in the infected midgut. The number of EdU-positive cells in *An. gambiae* mosquitoes was only about three to five times higher than baseline, however the exceptionally high counts of PH3-positive cells at that timepoint suggest that the epithelial response to infection in this species was delayed relative to the others.

Blood feeding is a major challenge for mosquito midgut physiology. To determine whether blood feeding affects epithelial dynamics, five-day-old mosquitoes were fed on bovine blood with EdU, and dissections were performed at the end of the blood digestion period (72 h PBM) (Fig. 2A). The blood meal triggered a modest but significant increase in the number of EdU-positive cells in the posterior midguts of *Ae. aegypti*, *Ae. albopictus*, *Cx. quinquefasciatus,* and *An. stephensi* (Fig. 2E). This increase in EdU incorporation (*Ae. aegypti P* < 0.0001, *Ae. albopictus P* < 0.0001, *Cx. quinquefasciatus P* = 0.0058, *An. stephensi P* < 0.0001, *An. gambiae P* < 0.0001, Welch’s t test, where *P* < 0.05 is significantly different) was not associated with a significant increase in PH3 counts in *Ae. aegypti* or *Cx. quinquefasciatus.* In the other mosquito species the mean counts of PH3-positive cells even decreased relative to the numbers in sugar-fed mosquitoes (Fig. S4A and B, *Ae. aegypti P* = 0.3617, *Ae. albopictus P* = 0.4706, *Cx. quinquefasciatus P* = 0.5629, *An. stephensi P* = 0.1736, *An. gambiae P* = 0.0075, Welch’s t test, where *P* < 0.05 is significantly different). Notably, the *An. gambiae* response to the blood meal was striking (Fig. 2B and E), with over 70% (SEM = 5.8) of the cells in the ROI incorporating EdU during the digestive period: a 13-fold increase relative to sugar-fed counterparts (Fig. 2E). The increase in EdU-positive cells was more modest in the other mosquito species, with counts being approximately 5 times higher in the blood-fed condition than in the sugar-fed baseline. In particular, *Ae. aegypti,* had a significant increase, going from 1.2% (SEM = 5.7) to 17.5% (SEM = 2.6).

All these results combined suggest that the mosquito midgut epithelium is capable of adjusting the levels of cell turnover in response to physiological challenges. The intensity and amplitude of the responses however, indicated potential differences between the responses to the same stimuli among the species studied. It is known however, that amongst mosquito species, different colony strains can have significant differences with respect to virus susceptibility, insecticide resistance, and other physiological parameters. Questions have been raised about the heterogeneity of laboratory strains bearing the same names in different institutions, their genetic variability over time, and the impact that this could have on reproducibility in scientific studies [45]. Furthermore, it is important to consider the applicability of findings obtained using mosquito strains that have been reared in laboratory conditions for several decades to field populations. To determine the level of variation that different mosquito strains of the same species could have in the rates of cell turnover in the midgut, we decided to study a range of strains, including long-kept laboratory strains, a newly established laboratory strain, and field-collected mosquitoes. Given that *Ae. aegypti* is one of the most popular laboratory models in vector biology, we took advantage of the availability of well-established lines with known susceptibility to viral infections, the strains known as “Liverpool” and “Rockefeller” and a strain less competent to Dengue virus, the “Orlando” strain [25, 46, 47]. Additionally, we included a newly established laboratory colony of *Ae. aegypti*, here named “Miami” strain; and field-collected adults from Guatemala (Fig. S5). Interestingly, all the *Ae. aegypti* strains that have been maintained in laboratory conditions for a long time showed less than 1% EdU-positivity among cells in the ROI. The Miami strain, in comparison, had 3.6% (SEM 4.7) and the field-collected mosquitoes from Guatemala had 2.1% (SEM 3.6). The differences in the percentage of cells incorporating EdU after blood feeding or infection were not statistically significant when compared between laboratory reared mosquitoes (Additional file 4: Fig. S4F-G), suggesting that these responses are conserved in the *Ae. aegypti* species. Considering this, we suggest that the nature of the epithelial responses to the bacterial challenges tested in this study can be generalized by species, although there may be small differences between strains or between laboratory versus field-collected mosquitoes. To answer questions regarding the kinetics and the nature of the response to each challenge, we therefore narrowed our focus to the two species that have the most medical relevance and are most commonly employed in studies of mosquito physiology: *An. gambiae* (G3 strain) and *Ae. aegypti* (Liverpool strain)*.*

### The posterior midgut epithelium of mosquitoes matures in the first day after emergence

To further characterize the maturation process, we supplemented the sugar diet of newly emerged *Ae. aegypti* and *An. gambiae* mosquitoes with a 24-h pulse of EdU (0–24, 24–48, or 48–72 h after emergence), immediately followed by dissection. To cumulatively measure EdU incorporation throughout adult maturation, we also maintained mosquitoes on EdU continuously for 72 h post-emergence prior to dissection (Fig. 3A-C). This allowed us to determine that most of the EdU accumulation occurring within the first 72 h of emergence was incorporated within the first 24 h (Fig. 3C). Interestingly, in *An. gambiae* mosquitoes, we failed to detect significant difference in the numbers of mitotic cells across the three timepoints (Fig. 3A, B), but still observed a large accumulation of EdU (Fig. 3C). In the first day after emergence, 21.7% (SEM 5.3) of the epithelial cells in the *An. gambiae* posterior midgut ROI were EdU-positive, suggesting that adult midgut maturation in this species entails a substantial endoreplication component uncoupled from progenitor cell proliferation. To confirm this hypothesis, we dissociated the posterior midgut into single cells, resuspended in flow cytometry buffer solution and quantified cell populations by ploidy using flow cytometry (Fig. 3D). The posterior midguts of newly emerged *An. gambiae* mosquitoes consisted chiefly of diploid, tetraploid and octaploid cells. Three days after emergence, these populations were no longer a majority and cells with higher ploidy (16C and 32C) were the most abundant (Fig. 3E). Finally, analysis of each cell population based on DNA content (Fig. 3F) demonstrated that more than 15% of the diploid, tetraploid and octaploid populations were EdU-positive. At least 40% of the 16C and more than 50% of the 32C cells were EdU-positive, confirming that those cells had undergone endoreplication since the time of emergence, as part of what we will hereafter refer to as the post-emergence gut maturation process.

In contrast to *An. gambiae*, the number of mitotic cells in the *Ae. aegypti* posterior midgut ROI was significantly higher (approximately 15x) at 24 h post-emergence than at either of the later timepoints (Fig. 3B). Combined with the high levels of EdU-positive cells in the ROI at this timepoint (36.6%, SEM = 5.2, Fig. 3C), this suggested intensive cell proliferation early after emergence in *Ae. aegypti.* Flow cytometry analysis confirmed that an initial pool of diploid cells at emergence was also notably expanded during the maturation period (Fig. 3E). Also during this period, the octaploid population was significantly reduced and the population of 32C cells increased, while the percentage of 16C cells remained relatively stable.

These results suggest that cells with higher ploidy are also generated in *Ae. aegypti* in the days after emergence. Further analysis of the percentage of EdU-positive cells within each cell population (Fig. 3F), also confirmed that most of the 64C and at least 30% of the 32C cells were EdU-positive. Altogether, the results show that, in the post-emergence maturation phase, the *Ae. aegypti* midgut undergoes epithelial proliferation*.* By contrast, at the timepoints evaluated, we did not detect significant numbers of mitotic cells in *An. gambiae.* Instead, in *An. gambiae,* EdU incorporation occurs with a concomitant transition of the cell population to cells of larger ploidy that compose the mature midgut epithelium.

### Epithelial dynamics are modulated in the posterior midgut in response to oral bacterial infection

The maintenance of the integrity of the midgut epithelial barrier has implications for mosquito fitness and vector competence. Cell turnover to replenish damaged cells has been shown to be essential for the maintenance of the midgut epithelium in Drosophila [48]. We leveraged the damaging ability of an oral entomopathogenic bacterium (*P. entomophila*) to test whether epithelial dynamics in mosquitoes adjust homeostatically to infection-mediated damage. In sugar-fed conditions we observed fewer than 5% of cells in the ROI incorporating EdU during a three-day period (Fig. 2D), and infection induced an increase of mitosis, compared to sugar-fed, which was detectable 72 h PI in *An. gambiae* and at 24, 48, and 72 h PI in *Ae. aegypti* (Fig. 4A, B)*.* Infection also resulted in an accumulation of EdU-positive cells, suggesting intense turnover. Assuming the turnover rate is stable in this condition this would mean that, during a 24-h period, approximately 1.6% of cells would be labelled with EdU. After infection with *P. entomophila*, the percentage of EdU-incorporating cells in the posterior midgut ROI of *An. gambiae* was 8% (SEM = 1.2) during each of the first two days PI, and 6% (SEM = 1.4) during the third day PI (Fig. 4C). Similarly, in *Ae. aegypti*, the basal turnover rate per-day could be calculated to be approximately 0.5% in unchallenged sugar-fed mosquitoes. After infection, this increased to 15.7% (SEM = 4) during the first 24 h, 12.7% (SEM = 4.7) the second day, and decreased to 0.34% (SEM = 0.09) on the third day, likely indicating the resolution of the infection. To confirm the different kinetics of the responses between *An. gambiae* and *Ae. aegypti*, we did an EdU/BrdU (5-Bromo-2’-deoxyuridine) pulse-chase assay where the mosquitoes were kept on sugar supplemented with EdU for 24 h after the infection and switched to BrdU thereafter (Additional file 5: Fig. S5A and Fig. 4D). In *An. gambiae*, similar numbers of EdU and BrdU-positive cells were found, consistent with a significant part of the cellular response occurring after the first 48 h PI, as suggested by the PH3 counts. An increased number of tetraploid cells was detected by flow cytometry in the *An. gambiae* midgut 72 h PI (Fig. 4E-F), which might reflect an increased number of progenitors in G2 phase. In *Ae. aegypti*, by contrast, the results from the EdU/BrdU pulse-chase showed a very high level of EdU incorporation, corresponding to the DNA synthesis from the first 24 h while BrdU incorporation, corresponding to events in the second- and third-day PI, was much more modest (Additional file 6: Fig. S6A). Flow cytometry results showed a significant increase in 32C, 64C and 128C cell populations in *Ae. aegypti* midguts 72 h PI, with 30–50% EdU-positivity among the cells in each population (Additional file 6: Fig. S6B). Overall, our results show that mosquito midguts can increase cell proliferation after an infection, probably to replenish lost cells.

### Blood feeding results in increased enterocyte ploidy, especially in *Anopheles gambiae*

We had previously observed that different mosquito species responded differently to the same blood source (Fig. 2B, E, and Additional file 3: Fig. S3). In addition, different mosquito species have different host ranges and preferences, suggesting that the epithelial response to blood feeding could be affected by blood source. To further explore this phenomenon, we fed *Aedes, Culex* and *Anopheles* mosquitoes with human, avian and artificial blood meals in addition to bovine blood. Our results show that, for most mosquito species, the extent of EdU incorporation in response to the blood meal differed significantly between blood sources (Fig. 5 and Additional file 7: Fig. S7). In *Ae. aegypti* (Fig. 5B)*,* human and bovine blood elicited similar amounts of EdU integration, whereas avian and artificial blood meal responses were less pronounced. In *Ae. albopictus* and *Cx. quinquefasciatus* mosquitoes (Additional file 7: Fig. S7), bovine blood elicited the strongest EdU incorporation response among all the blood sources tested. Interestingly, *An. stephensi* and *An. gambiae* mosquitoes presented distinctly different responses to the blood meal. In *An. stephensi*, the strongest response was to the artificial blood meal, being significantly different from those of *Aedes* and *Culex* mosquitoes (Fig. 5F). *An. gambiae* however, showed intense responses to human, bovine and artificial sources (Additional file 7: Fig. S7). Additionally, analyzing the responses by blood source allowed us to identify that, amongst all mosquito species, *An. gambiae* presented the strongest response to both the mammalian sources and to the artificial blood (Fig. 5C, D and F). The average response to the human blood meal across most species was ~ 20% EdU-positivity among cells in the posterior midgut ROI (Fig. 5C), with the exception of *An. gambiae*, which showed an average EdU-positivity of > 40%. Ingestion of bovine blood resulted in similar levels of EdU-positive cells in *Ae. aegypti, Ae. albopictus*, *Cx. quinquefasciatus* and *An. stephensi* (Fig. 5D)*.* It is important to note that *An. gambiae’*s response was, once again, especially high, with 72.8% (SEM = 5.8) of cells in the ROI EdU-positive. Finally, the response to avian blood was markedly muted in all species but *An. stephensi* (Fig. 5E)*.* Our results indicate that blood from the same source induces markedly different responses in different mosquito species. Given these findings, we sought to further characterize the nature of the response of the mosquito midgut epithelium to human blood in *An. gambiae* and *Ae. aegypti*. To this end, we dissected mosquitoes fed on human blood at early timepoints and quantified the levels of incorporation of EdU in the posterior midgut epithelium. *An. gambiae* mosquitoes showed a very rapid accumulation of EdU-positive cells in response to the blood meal, as early as two hours PBM (Fig. 6A, B). Twenty-four hours PBM, the percentage of EdU-positive cells was over 60% in the ROI and over 36% in the posterior midgut overall, resulting in a noticeable increase in ploidy in the larger EC population (Fig. 6F). This EdU incorporation was not correlated with stem cell proliferation as indicated by the absence of any important increase in mitotic activity (Fig. 6D). Accumulation of EdU-positive cells in *Ae. aegypti* (Fig. 6C) was much more modest than in *An. gambiae*, and the detection of mitotic cells in this species was also very low after the blood meal (Fig. 6E and Additional file 8: Fig. S8). In contrast to *An. gambiae*, in *Ae. aegypti,* the ploidy observed in the majority of the cells returns to a state similar to the pre-blood meal state (Fig. 6G). Overall, these results show that the mosquito midgut epithelium is an extremely dynamic tissue capable of changing the polyploid population of cells to favor larger ploidies when challenged.

## Discussion

### Progenitor proliferation is conserved in the adult midgut epithelium of *Aedes aegypti, Aedes albopictus*, *Anopheles gambiae* (*s.l*)*, Anopheles stephensi* and *Culex quinquefasciatus*

One significant finding of this study was that, in all the species we profiled, gut epithelial progenitors retained the ability to proliferate in the adult mosquito (Fig. 1 and Additional file 2: Fig. S2). This finding contrasts with the apparent lack of mitotic activity documented in blood fed *Cx. Quinquefasciatus* [31] by Okuda et al., and in *An. gambiae* (*s.l.*) of the G3 strain orally challenged by Janeh *et. al* with Sodium Dodecyl Sulfate (SDS) and *Serratia marcescens* [27]. This discrepancy is likely the result of methodological differences between our studies. Okuda et al. relied on transmission electron microscopy to survey the gut epithelium for evidence of proliferation and did not make use of PH3 antibody staining to probe the gut for mitotic nuclei, possibly allowing some mitoses to be overlooked. While Janeh et al. did employ a primary rabbit α-PH3 antibody, one possible explanation for the lack of PH3-positive cells in their preparations may lie in the methodology employed for immunostaining.

In *Ae. aegypti* and *An. gambiae* (*s.l.*), where we quantified PH3 and EdU incorporation in all five regions of the gut (Fig. 1), we found that epithelial dynamics were mostly confined to the midgut (proventriculus, anterior midgut, posterior midgut). While we did observe some PH3- and EdU-positive cells in the hindgut of *Ae. aegypti* (Fig. 1C, D), the PH3 cells were morphologically dissimilar from mitotic progenitors in the other regions (Fig. 1B), and we posit that PH3 in this case could be related to transcriptional regulation or that the EdU-positive cells we observed were the daughters of progenitors which migrated posteriorly from a hindgut proliferation zone at the posterior midgut junction, as previously documented in Drosophila [49]. Overall, our results corroborate prior reports of mitotic cells in the midgut epithelium of *Aedes, Culex* and *An. stephensi* mosquitoes, and we provide the first report of mitosis in the *An. gambiae* midgut epithelium. These findings should aid in the study of epithelial dynamics in all these species, facilitating a deeper understanding of vector-pathogen interactions.

### Post-emergence midgut maturation coincides with the post-emergence rise in juvenile hormone titer

All the mosquito species we examined exhibited a maturation period characterized by a heightened level of EdU incorporation over the first three days post-emergence (Fig. 2B). A closer examination of this phenomenon in *Ae. aegypti* and *An. gambiae* revealed that this activity was highest in the first 24 h post-emergence (Fig. 3A, C). In *Ae. aegypti*, we observed increased PH3-positive cell counts at this time (Fig. 3B), and a higher overall proportion of diploid cells by 3 days post-emergence (Fig. 3D, E), indicating that midgut maturation in this species entailed the proliferation of progenitors. In *An. gambiae*, by contrast, PH3-positive cell counts were low at all observed timepoints (Fig. 3B), and the proportion of diploid cells decreased while higher ploidies (32C, 64C) appeared (Fig. 3E), indicating that differentiation and endoreplication play a substantial role in the post-emergence maturation of the *An. gambiae* midgut, possibly with little or no accompanying mitosis. This decrease in diploid proportions was similar to what Maya-Maldanado et al*.* observed in the *An. albimanus* midgut in the first 24 h post-emergence [23]. Notably, the timing of peak EdU incorporation in *Ae. aegypti* and *An. gambiae* correlates with peak post-emergence juvenile hormone (JH) titers as measured in *Ae. Aegypti* [50], *An. Albimanus* [46], and Drosophila [47]. In Drosophila*,* JH serves as a post-mating anticipatory signal that stimulates ISCs to divide, yielding a larger gut to support the nutritional demands of reproduction [51]. JH has also been associated with endoreplication in insects [52] and, in *Ae. aegypti*, topical application of JH induces endoreplication in the fat body [53]. The mosquito midgut is known to be transcriptionally responsive to JH in the post-emergence phase [3, 54–58], generating a pool of trypsin and chymotrypsin transcripts in preparation for blood feeding. We hypothesize that, in addition to anticipatory effects on peptidase transcription, the post-emergence JH pulse prepares the mosquito midgut epithelium for its first blood meal by promoting some species-specific combination of proliferation and endoreplication.

### The magnitude of the blood feeding response is host-dependent

All mosquito species displayed an increased accumulation of EdU-positive cells in the midgut epithelium upon blood feeding (Fig. 2B and E). This phenomenon was especially marked in *An. gambiae* where 70% of cells in the ROI incorporated EdU in the absence of any significant increase in mitosis (Fig. 2E). This strongly suggests that blood feeding in this species prompts widespread endoreplication among mature ECs rather than prompting progenitor proliferation. This response may be secondary to the elevated ecdysone titers characterizing the vitellogenic phase of the mosquito gonotrophic cycle [59, 60]. In Drosophila, ecdysone is known to promote intestinal growth [61]. Interestingly, the magnitude of EdU accumulation in blood-fed guts of all species was found to be blood source-dependent (Fig. 5, Additional file 7: Fig. S7). In comparison to mammalian blood, avian blood stimulated markedly less EdU incorporation in *Cx. quinquefasciatus*, which has been documented to feed frequently from avian hosts in a natural setting [62] and in *An. gambiae* and *An. stephensi* (which, to our knowledge, have not) (Additional file 7: Fig. S7). The differential responses to avian versus mammalian blood could be accounted for by a difference in nutritional composition, heme content, the presence/absence of other host-specific factors, or some combination of these stimuli. Whether these factors impacted the nature of the response, as well as what effect each diet had on the size or composition of the gut’s microbial community, was not analyzed at this time. Some blood-source dependent microbial contamination might be suspected as the source of the variation in response to different animal blood types, We consider this explanation unlikely, however, as phenotypes were consistent across three replicates, and the artificial blood meal, SkitoSnack [63], which was freshly formulated in the lab from dry ingredients immediately before each feed, and was therefore unlikely to harbor significant microbial contamination, induced a robust accumulation of EdU-positive cells in the midguts of both *Anopheles* species. The SkitoSnack formulation comprises ATP, inorganic ions, glucose, chicken yolk (a lipid/protein source), bovine serum albumin (a protein source) and bovine hemoglobin (Fig. 5A, F). We therefore conclude that the differential amount of DNA synthesis stimulated in the *Anopheles* gut epithelium in response to avian versus mammalian blood reflects either differing nutrient or heme concentrations. In Drosophila, nutrient levels in the gut lumen are sensed via insulin signaling, resulting in altered ISC activity that adaptively re-sizes the gut to optimize absorption [12, 64]. Insulin signaling in Drosophila is also sufficient to increase ploidy in differentiating ECs [65]. One key function of polyploidization is the increase in biosynthetic capacity in metabolically active cells [66, 67], the progression of ECs to higher ploidies could help ECs increase the production of digestive enzymes for efficient digestion of the blood meal. Differing nutrient levels may also induce variable levels of microbial proliferation, with implications for epithelial cell dynamics. Heme may also act directly, stimulating epithelial repair by stressing the gut through the production of ROS which, in Drosophila, activates ISC activity [15, 68, 69]. Alternatively, a heme response may also be microbe-mediated; in *Ae. aegypti*, heme has been shown to activate PKC in a dose-dependent manner, lowering epithelial ROS and allowing the increased proliferation of gut microbes [70].

### DNA synthesis in the midgut: distinguishing between progenitor proliferation, EC differentiation and endocycling in mature ECs

EdU incorporation into the nuclei of different cells in the mosquito midgut epithelium could represent three types of cell cycle: one round of DNA replication preceding mitosis in progenitors (canonical cell cycle with mitosis), endoreplication during the differentiation of progenitors into mature polyploid ECs (canonical postmitotic endoreplication to achieve ECs of greater ploidy), or endoreplication-associated cell cycle reentry in mature ECs that exited a replicative cycle for some time prior to the reentry event. Distinguishing between these modes of EdU incorporation can present a challenge. Mitosis may be affirmatively demonstrated by the presence of PH3-positive cells or implied by the presence of EdU-positive diploid cells. An increase in the proportion or number of diploid cells may also suggest proliferative activity – with the caveat that the disproportionate loss of polyploid cells may also be responsible for such a change. A shift in proportion away from diploid cells suggests the differentiation of progenitors into ECs, possibly achieved by symmetric division favoring EC formation (progenitor cell division followed by the differentiation of both daughter cells). Demonstrating the absence of mitosis, however, is considerably more difficult. Since mitosis comprises only a short portion of the canonical cell cycle (40 min out of several hours, depending on the cell type), a proliferative phase may pass unremarked if an experiment is timed either too early or too late. Any resulting EdU-positive EC daughters are, to appearance, indistinguishable from progenitors that have differentiated without dividing, or from ECs that have activated endoreplication in their maturity to cycle to a higher ploidy.

In the absence of genetic lineage tracing techniques used easily in other model species, or any means to ablate proliferation in the mosquito midgut without compromising cells’ ability to replicate DNA, it may prove challenging to conclusively distinguish which phenomenon accounts for the accumulation of EdU-positive cells. However, some of our findings strongly suggest that, in some conditions mature polyploid ECs, which are conventionally thought to be entirely post-mitotic/post-replicative, can reenter the cell cycle to further increase their ploidy. We hypothesize that this is a mechanism adopted by the *An. gambiae* species to digest the blood meal rapidly and efficiently. In representative images of human blood-fed *An. gambiae* posterior midguts, 3 days PBM, many approached a 100% level of EdU-positive cells in the ROI, with no similar accumulation occurring in their sugar-fed counterparts (Fig. 2E). If mature cells did not integrate new DNA, the lack of DAPI-positive/EdU-negative ECs in these images could only otherwise be explained by the loss and replacement of most or all mature ECs in the ROI. On close inspection, some large ECs appeared very bright with EdU, while others were only slightly EdU-positive (Fig. 6A), indicating that, in some cases, the EdU integration we observed occurred in cells that were already polyploid prior to blood feeding/EdU supplementation. Flow cytometry demonstrated that new populations of high-ploidy EdU-positive cells (128C, 256C) appeared within two hours PBM. It is improbable that a newly differentiating progenitor could reach such a high level of ploidy in so short a time (Additional file 8: Fig. S8). Flow cytometry further demonstrated cell populations in blood-fed *An. gambiae* shifting from ~ 75% < 32C to ~ 60% > 32C over three days (Fig. 6F). While a shift of this magnitude might theoretically be caused by extensive cell loss and replacement, sampling at multiple timepoints failed to uncover a wave of PH3-positive cells sufficient to support such turnover (Fig. 6D). The extant populations of diploid and tetraploid cells (potentially progenitors in G2 phase) in the sugar-fed posterior midgut might be sufficient to generate the expanded proportions of high-ploidy ECs through differentiation alone, in combination with a disproportionate loss of 16C cells. However, neither the diploid nor tetraploid populations were substantially depleted at 3 days PBM. Rather the increased proportions of 32C, 64C, and 128C cells were almost perfectly balanced by the reduction in the proportion of 16C cells (Fig. 6F). These results are most readily explained by the activation of endoreplication in the formerly quiescent 16C population. This inference, if correct, contrasts with a previous report in Drosophila where Xiang et al. found that, in the context of infected midguts, endoreplication was exclusively confined to EBs and newly differentiated ECs(20). In the future, it will be interesting to learn whether the higher ploidy in the blood-fed *An. gambiae* midgut epithelium persists, and whether multiple blood feedings alter the endoreplication reentry response.

## Conclusions

In summary, progenitor cells in the adult midgut epithelium of *Ae. aegypti, Ae, albopictus, An. gambiae (s.l.), An. stephensi,* and *Cx. Quinquefasciatus* retain the ability to proliferate. This finding is especially noteworthy in *An. gambiae* as prior studies had suggested a lack of miotic activity in the midgut epithelium of this species. Our results demonstrate a conserved maturation period in the adult midgut epithelium of these mosquitoes, characterized by heightened DNA synthesis. In addition, progenitors respond to insults to the midgut epithelium such as infectious damage, suggesting that homeostatic pathways control the structure of the adult midgut. This discovery opens new avenues for the development of vector control strategies that target the molecular pathways guiding this process.

The blood-feeding event also stimulates increased DNA synthesis. By demonstrating that the response of the mosquito midgut epithelium to blood feeding is host-dependent and that the magnitude of the response varies depending on the mosquito species, we highlight the variability of the baseline response to the blood meal alone. In *An. gambiae* mosquitoes, this response is predominantly through endoreplication rather than progenitor proliferation, displaying a novel and characteristic cellular response to the blood meal that should be studied further to fully comprehend the cellular response occurring in the epithelium. These factors will be crucial to consider in future studies involving viral and parasitic infections.

Overall, these findings contribute to a better understanding of the dynamics of the mosquito midgut epithelium, providing a detailed baseline for studying the mosquito midgut response to pathogens, as well as for the study of the roles of hormonal and nutritional factors regulating the epithelial cell proliferation, endoreplication, and differentiation. By implementing standardized methods and counts across different species and strains our study also provides uniformity and precision that enabled a deeper and more reliable understanding of midgut epithelial cell dynamics.

## Methods

### Animal rearing

#### Laboratory-reared mosquitoes

*Ae. aegypti* (Liverpool, Rockefeller and Orlando strains), *Cx. quinquefasciatus* (Johannesburg strain, Catalog No. NR43025), *An. gambiae* (*s.l.*, G3 strain, Catalog No. MRA-112) and *An. stephensi* (STE2 strain, Catalog No. MRA-128) were obtained from BioDefense and Emerging Infections (BEI) Malaria Research and Reference Reagent Resource Center (MR4), Centers for Disease Control and Prevention (CDC), Atlanta, Georgia, USA. *Ae. albopictus* and a newly established laboratory line of *Ae. aegypti* collected in Florida, USA (here named “Miami” strain), were kindly provided by Alexander Ciota. Insectary conditions were 28 °C and relative humidity of 80% with a photoperiod of 12:12 light: dark photocycle with a 30-min dawn and dusk period. Adults were fed ad libitum on a 10% sucrose solution.

#### Field mosquitoes

*Ae. aegypti* mosquitoes were collected in the department of Chiquimula, Guatemala for three consecutive days in collaboration with the personnel of the Vector-Control team of the local Health Center (coordinates of locations are included in Table 1). Backpack aspirators (Prokopack, John W. Hock Company, FL, USA) were used to collect adult mosquitoes in fifty different domiciliary locations. Larvae and pupae from water reservoirs at the same sites were also collected. After each collection, adult mosquitoes were offered 10% sucrose with the nucleotide analog EdU, 100 µM, for 24 h before dissection. A total of 207 adult mosquitoes were obtained. All collected pupae (*n* = 93) and larvae (*n* = 44) were transferred to the laboratory facility at Del Valle de Guatemala University, where they were maintained at 27 °C, relative humidity of 70 ± 10%, and 12:12 photoperiod. After emergence, mosquitoes were offered 10% sucrose for five days, and then maintained on 10% sucrose/100 µM EdU for 72 h before dissection. Genotypification of each mosquito, using mosquito legs as DNA source, was performed by polymerase chain reaction (PCR) as previously described [71]. Only female mosquitoes positively identified as *Ae. aegypti* by PCR (*n* = 142 for mosquitoes collected as adults, and *n* = 46 for mosquitoes collected as larvae and pupae) were included in the study.

**Table 1:** GPS coordinates of the collection sites for *Ae. aegypti* mosquitoes in Chiquimula, Guatemala

Time	Name	Latitude	Longitude	Elevation
2021–08-17T15:12:36Z	BC1	14.814671	-89.551386	455.541931
2021–08-17T16:02:51Z	BC2	14.811919	-89.549925	444.658569
2021–08-17T16:33:57Z	BC3	14.81178	-89.548463	442.898682
2021–08-18T18:00:04Z	BC5	14.813715	-89.551138	452.981598
2021–08-18T19:53:52Z	BC6	14.813709	-89.551112	450.098572
2021–08-18T19:56:59Z	001	14.813793	-89.550811	453.177979
2021–08-18T20:22:29Z	BC7	14.813429	-89.550118	463.136993
2021–08-18T20:51:31Z	BC8	14.812858	-89.550273	445.828003
2021–08-19T14:33:11Z	BC10	14.812833	-89.551432	448.616516
2021–08-19T14:59:20Z	BC11	14.812839	-89.550952	441.364746
2021–08-19T15:25:54Z	BC12	14.812886	-89.550993	439.965424
2021–08-19T15:42:08Z	BC13	14.812885	-89.551142	442.725739
2021–08-19T15:57:41Z	BC14	14.812938	-89.551139	443.377136
2021–08-19T16:12:12Z	BC15	14.813231	-89.550995	447.766449
2021–08-17T15:57:02Z	AC1	14.8122	-89.521152	353.312073
2021–08-17T16:02:30Z	AC2	14.811474	-89.520893	354.271423
2021–08-17T16:05:59Z	AC3	14.811429	-89.52097	355.055756
2021–08-17T16:21:25Z	AC4	14.811453	-89.52098	354.926697
2021–08-17T16:34:05Z	AC5	14.807722	-89.522256	360.126465
2021–08-17T16:51:03Z	AC6	14.807729	-89.522243	362.277344
2021–08-17T16:53:49Z	AC7	14.805692	-89.527568	372.142975
2021–08-17T17:04:25Z	AC8	14.805425	-89.529005	375.76593
2021–08-17T17:31:31Z	AC9	14.805387	-89.528923	374.879822
2021–08-17T17:44:56Z	AC10	14.805498	-89.528682	386.457703
2021–08-17T20:18:40Z	AC11	14.80542	-89.528809	386.930328
2021–08-17T20:21:13Z	AC12	14.805229	-89.53003	389.340912
2021–08-17T20:28:24Z	AC13	14.805096	-89.530434	388.454681
2021–08-17T20:50:29Z	AC14	14.80513	-89.53037	388.705505
2021–08-18T17:55:25Z	AC16A	14.806575	-89.525596	371.095612
2021–08-18T18:06:34Z	AC17A	14.8061	-89.525894	390.181641
2021–08-18T20:17:28Z	AC18A	14.806057	-89.526149	389.054626
2021–08-18T20:27:22Z	AC19A	14.806002	-89.526352	382.111176
2021–08-18T20:44:17Z	AC20A	14.805988	-89.526612	381.7146
2021–08-19T14:09:24Z	AC22A	14.805644	-89.527844	378.047577
2021–08-19T14:25:35Z	AC23A	14.805635	-89.528	376.168335
2021–08-19T14:31:33Z	AC24A	14.805575	-89.528062	378.696014
2021–08-19T14:53:14Z	AC25A	14.805554	-89.528155	375.609436
2021–08-19T15:07:31Z	AC26A	14.805485	-89.528305	374.947998
2021–08-19T15:19:00Z	AC27A	14.805489	-89.528322	370.346405
2021–08-19T15:40:29Z	AC28A	14.805529	-89.528362	376.223999
2021–08-19T15:50:49Z	AC29A	14.805542	-89.528361	371.708527
2021–08-19T15:58:31Z	AC30A	14.805362	-89.529007	376.675232
2021–08-19T16:59:28Z	AC31A	14.805373	-89.528945	374.462463
2021–08-19T17:22:44Z	AC32A	14.805292	-89.529152	369.845764
2021–08-19T20:25:27Z	AC33A	14.805282	-89.529342	372.355011
2021–08-19T20:31:23Z	AC35A	14.805261	-89.529362	373.654114
2021–08-19T20:39:03Z	AC34A	14.805195	-89.529552	375.56189
2021–08-19T20:40:51Z	AC36A	14.805213	-89.52968	374.4245
2021–08-19T20:53:43Z	AC38A	14.804997	-89.530605	379.238129
2021–08-19T21:07:55Z	AC39A	14.805089	-89.530153	381.182953

#### Mosquito rearing for sugar-fed and maturation conditions

To characterize the cellular dynamics of the midgut, five-day-old mosquitoes were fed on 10% sucrose/100 µM EdU for 72 h and this condition was used as the baseline for comparison with newly emerged mosquitoes, as well as with same-age mosquitoes either blood-fed or infected. While all groups of sugar-fed, blood-fed, and infected mosquitoes were reared and dissected synchronously, maturation experiments were carried out independently due to the need to collect adults who emerged during a six-hour window, to avoid age variability within this group. To study newly emerged mosquitoes, pupae were transferred to water containing a 100 µM solution of EdU and continuously reared for 72 h post-emergence on 10% sucrose/100 µM EdU, prior to dissection (Fig. 2A). To more closely characterize the proliferative state, DNA synthesis, and ploidy profiles in *Ae. aegypti* and *An. gambiae* midgut epithelial cells during each of the first three days post-emergence (0–24-h, 24–48-h, 48–72-h), as well as across the entire maturation period (72-h cumulative), we either transferred pupae to water containing 100 µM EdU and reared emergent adults on 10% sucrose/100 µM EdU (0–24-h and 72-h cumulative samples), or we provided adult mosquitoes with a 24-h pulse of 10% sucrose/100 µM EdU (from 24–48 h, or 48–72 h) prior to dissection. In every case, at least three biological replicates were performed with 10–15 mosquitoes each.

#### Blood feeding

Mature adults (at least 6 days old) were starved for at least 3 h before a blood meal. Bovine blood (Lampire, PA, USA) was used for blood meals unless otherwise stated. For experiments to compare the effects of blood source, we used red blood cells Citrate–phosphate-dextrose solution with adenine (CPDA) O + from male donors mixed with human plasma from normal, healthy male donors (cat HP1055RPM1835) (Valley Biomedical, VA, USA), avian blood (rooster, Lampire) and the SkitoSnack blood-replacement formulation developed by Gonzales, et al. [63]. Bovine and avian blood was treated with sodium citrate as anticoagulant and all blood meals were supplemented with 100 µM EdU (final concentration), to track DNA synthesis. In every replicate, the same batch of blood from each source was used to feed all mosquito species. Unless otherwise stated, mosquitoes were maintained on 10% sucrose/100 µM EdU after the blood meal and midguts were dissected after 72 h. For each feeding, at least three biological replicates were performed with 10–15 mosquitoes each. For the species of special interest (*Ae. aegypti* and *An. gambiae*), intermediate time points of 0.5, 2, 6, 12, 24 and 48 h PBM were also collected.

#### Oral infection

Three doses of the bacterium *Pseudomonas entomophila* were tested (OD_600_ 25, 50 and 100) to establish an infection protocol that would induce a robust response in mosquitoes. Optimal results were observed with an OD_600_ of 100 (Fig. S1A), and therefore that dose was used for all oral infection experiments. For infections, a cotton ball was soaked in a 1:1 mixture of 20% sucrose/200 µM EdU and concentrated bacterial culture (OD_600_ 200) and offered to 5-day-old adult mosquitoes that had been starved for at least 3 h. Unless otherwise stated, infected mosquitoes were kept on 10% sucrose/100 µM EdU for 72 h. For the species of special interest (*Ae. aegypti* and *An. gambiae*), intermediate timepoints of 24- and 48-h PI were also collected. Additionally, an experiment with mosquitoes infected and maintained on 10% sucrose/100 µM EdU for 24 h was switched to 10% sucrose containing 1 µM of the nucleotide analog BrDU and maintained on 10% sucrose/1 µM BrDU for 48 h, until the time of dissection (Additional file 5: Fig. S5). For each infection, at least three biological replicates were performed with 10–15 mosquitoes each.

### Cell proliferation and DNA synthesis labeling

Quantification of mitoses in whole-midgut tissues was performed by PH3 labeling as previously described [25]. Briefly, adult female mosquitoes were dissected in ice-cold PBS. Midguts were fixed in phosphate buffered saline (PBS) with 4% paraformaldehyde (Electron Microscopy Sciences, PA, USA) for 30 min at room temperature and stored in PBS at 4 °C until processing. For permeabilization, tissues were incubated in PBS with 0.3% Triton X-100 for 15 min at room temperature, and then blocked with PBS, 0.1% Tween 20, 2.5% bovine serum albumin (BSA) and 10% normal donkey serum (Jackson Laboratories, PA, USA) for at least 30 min at room temperature. All samples were incubated with primary rabbit anti-PH3 antibody (1:500) (Merck Millipore, Darmstadt, Germany). After washing 3 times for 20 min each in PBS, 0.1% Tween 20, 0.25% BSA, samples were incubated with secondary donkey anti-rabbit antibody conjugated with Alexa Fluor 555 or 647 (1:2000) (Thermo Fisher Scientific, MA, USA) for at least two hours at room temperature. Samples from mosquitoes fed with EdU were additionally subjected to a reaction from the Click-iT™ EdU Cell proliferation kit for imaging with Alexa Fluor 488 or 555 (ThermoFisher Scientific) as per the manufacturer’s instructions in between the permeabilization and blocking steps. Finally, DNA was counterstained with 4′,6-diamidino-2-phenylindole (DAPI) (1 µg/ml) (Sigma, MO, USA) and guts were mounted for confocal microscopy in Citifluor AF1 antifade medium (Electron Microscopy Sciences). Imaging was performed on a Zeiss LSM 700 fluorescent/confocal inverted microscope (Zeiss, Oberkochen, Baden-Wurttemberg, Germany).

### Flow cytometry

Mosquito posterior midguts were dissected in ice-cold PBS, fixed and permeabilized as previously described. Click-iT reactions for EdU labeling were also performed as described for imaging. Pools of 8 mature guts, or 20 to 40 immature guts, were transferred to 100 µL of solution containing 1 mg/ml of elastase (Sigma-Aldrich) for dissociation. Additionally, for each mosquito species, pupal brains (5 to 8 per pool) were treated in the same manner to obtain diploid controls. After a 5-min incubation at 37 °C, the tissue was triturated using p200 pipette tips by pipetting vigorously for periods of 60 s per sample, at least two times, until there were no visible fragments of tissue, and a single-cell suspension was obtained. All samples were diluted to a final volume of 800 µL in PBS, 1% BSA, 1 µM ethylenediaminetetraacetic acid (EDTA) and 1 µL/mL DAPI. After gently vortexing, samples were loaded onto the Attune flow cytometer for flow cytometry analysis, using a laser configuration of a violet laser (VL, 405 nm) with six bandpass (BP) filters and a blue laser (BL, 488 nm) with three bandpass filters. The detection of DAPI was performed using VL1 (Emission filter 450/40), EdU in green using BL1 (Emission filter 530/30), EdU in red using BL2 (Emission filter 647). A flow rate of 100 to 500 µL/second was used for sample acquisition and a minimum of 5,000 events gated as “non doublets” were acquired per sample. The gating strategy, adapted from Nandakumar et al. [72], is graphed in Additional file 9: Fig. S9. Briefly, all cells were plotted on forward versus side scatter (FSC vs. DAPI-A) and gated to eliminate debris. Subsequently, “non-debris” were plotted on DAPI(DNA)-H vs DAPI(DNA)-A (voltage pulse area vs. height) to eliminate doublets. All events in the non-doublet gate were further subjected to DNA/EdU content analysis.

### Statistical analysis

Mann–Whitney test was utilized where comparisons were made between 2 groups without making any distributional assumptions (Figs. 1 and 2D-F). Where more than 2 treatments are analyzed, a Brown-Forsythe and Welch ANOVA (analysis of variance) test for multiple comparisons was utilized. Dunnett’s T3 multiple comparisons test for multiple comparisons was used in these cases to analyze differences between groups in 3B, C, and F; 4B and C; and 6B and C. In datasets showing cell counts through different days, the result of the Dunnett’s T3 tests are shown as *, **, and *** respectively indicating *P* values of < 0.05, < 0.001, and < 0.001. Where percentages of EdU-positive cells were analyzed in each cell group based on ploidy size, groups were assigned IDs as follows: 2C = a, 4C = b, 8C = c, 16C = d, 32C = e, 64C = f, 128C = g and the letters at the top of each bar indicate which groups are significantly different. All statistical analyses were performed using GraphPad Prism Software version 8.02 (GraphPad Software, CA, USA).

### Supplementary Information

**Additional file 1: Supplemental Figure 1.** Bacterial infection protocols. (A) Selection of an optimal dose of bacteria to use for all experiments was done by quantifying the percentage of EdU-positive cells resulting from *Pseudomonas entomophila *infections in *Aedes aegypti *using OD_600_ of 25, 50 and 100. An OD_600_ of 100 was selected due to the more consistent increase of EdU-positive cells. Results are from at least three biological replicates. Statistics: Brown-Forsythe and Welch ANOVA tests for multiple comparisons, Alpha 0.05. (B) Upper timeline shows the protocol for continuous EdU incorporation in the infection experiments: Mature, 5-day-old females were infected with a sucrose-baited solution containing *P. entomophila *and EdU, thereafter maintained on sucrose/EdU and dissected at 24- or 72-hours post-infection (PI). Guts were treated with a Click-iT cocktail to label EdU (green) and stained with an anti-PH3 antibody (red) and DAPI (blue). This protocol was used to infect *Ae. aegypti, Aedes albopictus, Culex quinquefasciatus, Anopheles gambiae *and* Anopheles stephensi.* In a second set of experiments (bottom timeline), used only with *Ae. aegypti* and *An. gambiae, *mosquitoes were kept on sucrose/EdU for 24 hours (including the time of infection), then switched to sucrose/BrdU.

**Additional file 2:** **Supplemental Figure 2.** Gating strategy for Flow Cytometry analysis. (A) All events were plotted on forward versus side scatter (FSC vs. DAPI-A) and gated to eliminate debris. This was the primary gate (H1) which included non-debris, stained events. (B) Stained events included in the primary gate (H1) were plotted on DAPI(DNA)-H vs DAPI(DNA)-A (voltage pulse area vs. height) to eliminate doublets. This secondary gate was called “non-doublets”, five thousand events were required in this gate for a sample to be included in the analysis. (C) All events in the non-doublet gate were then plotted as EdU fluorescence (y axis) vs DNA content (x axis) and gates of EdU-positive and EdU-negative events were established using samples with no EdU treatment to set up negative control values. (D) For each species, larval brains were dissected (5 per sample) and stained with DAPI for diploid controls. Overlapping histograms of the diploid control and the midgut epithelial cells of sugar-fed samples were used to identify the placement of the diploid population amongst the ones in the midgut epithelium. Cell count on the Y axis is a normalized value relative to the total cell count. Each experiment was performed in at least three biological replicates.

**Additional file 3: Supplemental Figure 3.** PH3- and EdU-positive cell counts normalized to total cell number per gut region. Five-day-old females were kept on 10% sucrose with 100 µM EdU for 72 hours. Full guts were dissected on ice-cold PBS and immune-stained to label mitosis using PH3 antibody (1:500). Counts of DAPI+ nuclei were used to estimate total cell number per region (A). *Aedes aegypti* guts had approximately double the quantity of cells in the *Anopheles gambiae *guts. PH3-positive cells presented in percentage are shown in (B), and EdU-positive cells are presented in (C). Values on top indicate mean values, and error bars are SEM. Mann-Whitney tests were performed between species and *P* values < 0.05 were considered significantly different. Each experiment was performed in biological triplicates. (D) Individual cell counts of EdU positive cells in each biological replicate, presented by total number of cells per region in *Ae. aegypti* and *An. gambiae, *show that there was no batch effect detectable when*.*

**Additional file 4: Supplemental Figure 4.** PH3-positive cells were observed in *Aedes, Culex *and *Anopheles* mosquitoes. Fixed tissue from eight-day-old females that had either been sugar-fed, blood-fed or infected, and maintained on 10% sucrose with 100 µM EdU for 72 hours, was treated uniformly across species, through permeabilization, Click-it, blocking and antibody staining protocols. Primary antibody, rabbit anti-PH3 (1:500) and secondary antibody, goat anti-rabbit Alexa 555 (1:2000), were used for all samples. Quantification of PH3-positive cells per posterior midgut in sugar-fed (A), blood-fed (B), and infected (C) mosquitoes was done in *Aedes aegypti, Aedes albopictus, Culex quinquefasciatus, Anopheles gambiae *and *Anopheles. stephensi.* Values on top indicate mean values, and error bars are SEM. Mann-Whitney tests were performed between species and *P* < 0.05 was considered significantly different. Each experiment was performed in biological triplicates. Representative images of PH3- and EdU-positive cells for each species, under infection conditions, are shown in (D). (Scale bar = 50 µm) and white arrows indicate yellow (double-stained) cells.

**Additional file 5: Supplemental Figure 5.** Percentages of EdU-positive cells in *Aedes, Culex *and *Anopheles* mosquitoes changed with different physiological conditions. Fixed tissue from either three days old (maturation group, Mat) and eight-day-old females that had either been sugar-fed (SF), blood-fed (BF) or infected (Inf), and maintained on 10% sucrose with 100 µM EdU for 72 hours, was treated with a Click-it reaction to visualize cells that had incorporated EdU through the synthesis of DNA. Results are from at least three biological replicates. Values on top indicate mean values and error bars are SEM. Statistics: one-way ANOVA, *P* <0.001.

**Additional file 6: Supplemental Figure 6.** The midgut epithelium of adult female mosquitoes shows conserved responses under sugar-fed, blood-fed and infected conditions in different strains of *Aedes aegypti*. (A) Five-day old female *Ae. aegypti* of the Liverpool, Rockefeller, Orlando and Miami strains were maintained for 72 hours on a diet of 10% sucrose supplemented with EdU for the sugar-fed group. Similarly, five-day-old females were either blood-fed on bovine blood or infected with *P. entomophila *and maintained on 10% sucrose with EdU for 72 hours prior to dissection. Guts were treated with a Click-iT cocktail to label EdU (green) and stained with an anti-PH3 antibody (red) and DAPI (blue), (scale bar = 50 µm). Additional *Ae.* *aegypti* were field collected in the department of Chiquimula, Guatemala in adult and larval stages (B). The adults were dissected 24 hours after collection to obtain representative images of the state of gut epithelial dynamics in the wild, and larvae were transported to the insectary at Del Valle de Guatemala University to be maintained until 5 days post-emergence. Representative images of the midgut epithelium of field-collected mosquitoes are shown in (C), including mosquitoes collected as adults (top) and larvae (bottom). Adults were classified by gut content/gravidity as “blood-fed”, “semi-gravid” or “gravid”. Larvae were reared to adulthood under laboratory conditions. Total counts of PH3-positive cells on these groups are shown in (D). Values on top indicate mean values, and error bars are SEM. Quantifications of EdU-positive cells in the region of interest of all laboratory-reared mosquitoes (including those reared from field-collected larvae) are shown in (E). Consistent with prior results, Liverpool, Rockefeller and Orlando strains showed average numbers of EdU-positive cells below 1%. Miami strain and field-collected mosquitoes from Guatemala showed significantly higher numbers. The percentages of EdU-positive cells were not significantly different between blood-fed (F), and infected mosquitoes (G) from different *Ae. aegypti* strains, suggesting that the amplitude of epithelial responses to these stimuli are broadly conserved within the species. Results are from at least three biological replicates. Values on top indicate mean values, and error bars are SEM. Statistics: Mann-Whitney test; *, **, and *** respectively indicate *P* values of <0.05, <0.001, and <0.001. Field-collected mosquitoes from Guatemala showed low levels of EdU incorporation (H), consistent with the numbers observed in sugar-fed mosquitoes over a 24-hour period (Fig. 3C), all showing less than 1% of EdU-positivity in the region of interest.

**Additional file 7: Supplemental Figure 7.** EdU and BrdU incorporation in orally infected mosquitoes through time and after three days by ploidy. Fixed midguts from eight-day-old females that had been infected with *Pseudomonas entomophila* and maintained on 10% sucrose with 100 µM EdU for 24 hours and, subsequently, on 10% sucrose with 1 µM BrdU for 48 hours, were treated with a Click-iT reaction for EdU (green) and immunostaining for BrdU (red) (A). Different cell populations, based on DNA content, had incorporated EdU after 72 hours of feeding with sucrose supplemented with EdU (B). The upper row presents the percentages of EdU-positive cells per cell population in sugar-fed mosquitoes (unchallenged) and the lower row presents percentages of EdU-positive cells per cell population in *P. entomophila*-infected mosquitoes. Samples consisted of pools of 8 posterior midguts, *n*=9, from at least three biological replicates.

**Additional file 8: Supplemental Figure 8. **EdU incorporation in mosquitoes blood-fed with different blood-sources. Five-day-old female mosquitoes were blood-fed on human, bovine or avian blood, as well as on the artificial diet SkitoSnack (all supplemented with 100 µM EdU) and then maintained on 10% sucrose with 100 µM EdU for 72 hours (A-E). Different blood sources resulted in different responses from the same mosquito species. Values on top indicate mean values, and error bars are SEM. Mann-Whitney tests were performed between species and *P* < 0.05 was considered significantly different. Each experiment was performed in biological triplicate.

**Additional file 9: Supplemental Figure 9.** EdU incorporation in the midgut of *Anopheles gambiae *and *Aedes aegypti* after blood feeding by ploidy. (A) In the epithelium of *An. gambiae, *the number of larger ploidy cells showing incorporation of EdU is high even at two hours after the blood meal. The percentage of EdU-positive cells in all cell populations is significantly higher by the end of the digestive process. (B) In *Ae. aegypti, *the overall quantity of EdU-positive cells is less than in *An. gambiae *(Fig. 6C), but there is also a significant incorporation of EdU in all the cell populations. Samples consisted of pools of 8 posterior midguts, *n*=9 samples per condition, from at least three biological replicates.

## Data Availability

The datasets used and/or analyzed during the current study are available from the corresponding author on reasonable request.

## Abbreviations

2C: Diploid
4C: Tetraploid
8C: Octaploid
ANOVA: Analysis of variance
ATP: Adenosine triphosphate
BEI: BioDefense and Emerging Infections
BSA: Bovine Serum Albumin
BrdU: 5-Bromo-2’-deoxyuridine
CDC: Centers for Disease Control and Prevention
CPDA: Citrate–phosphate-dextrose solution with adenine
DAPI: 4′,6-Diamidino-2-phenylindole
DNA: Deoxyribonucleic acid
EB: Enteroblast
EC: Enterocyte
EE: Enteroendocrine
EEP: Enteroendocrine progenitor
EDTA: Ethylenediaminetetraacetic acid
EdU: 5-Ethynyl-2'-deoxyuridine
EGFR: Epidermal growth factor receptor
ERK: Extracellular signal-regulated kinase
ISC: Intestinal stem cell
JH: Juvenile Hormone
OD_600_: Optical density at 600 nm
PCR: Polymerase chain reaction
PH3: Phosphorylated Histone-3 (PH3)
PBM: Post blood-meal
PBS: Phosphate buffered saline
PI: Post-infection
ROI: Region of interest
SEM: Standard error of the mean
