# Supplementary figures and images for: The midgut epithelium of mosquitoes adjusts cell proliferation and endoreplication to respond to physiological challenges
Source: BMC Biol. 2024 Jan 29;22:22. doi: 10.1186/s12915-023-01769-x (PMC10823748; doi:10.1186/s12915-023-01769-x)

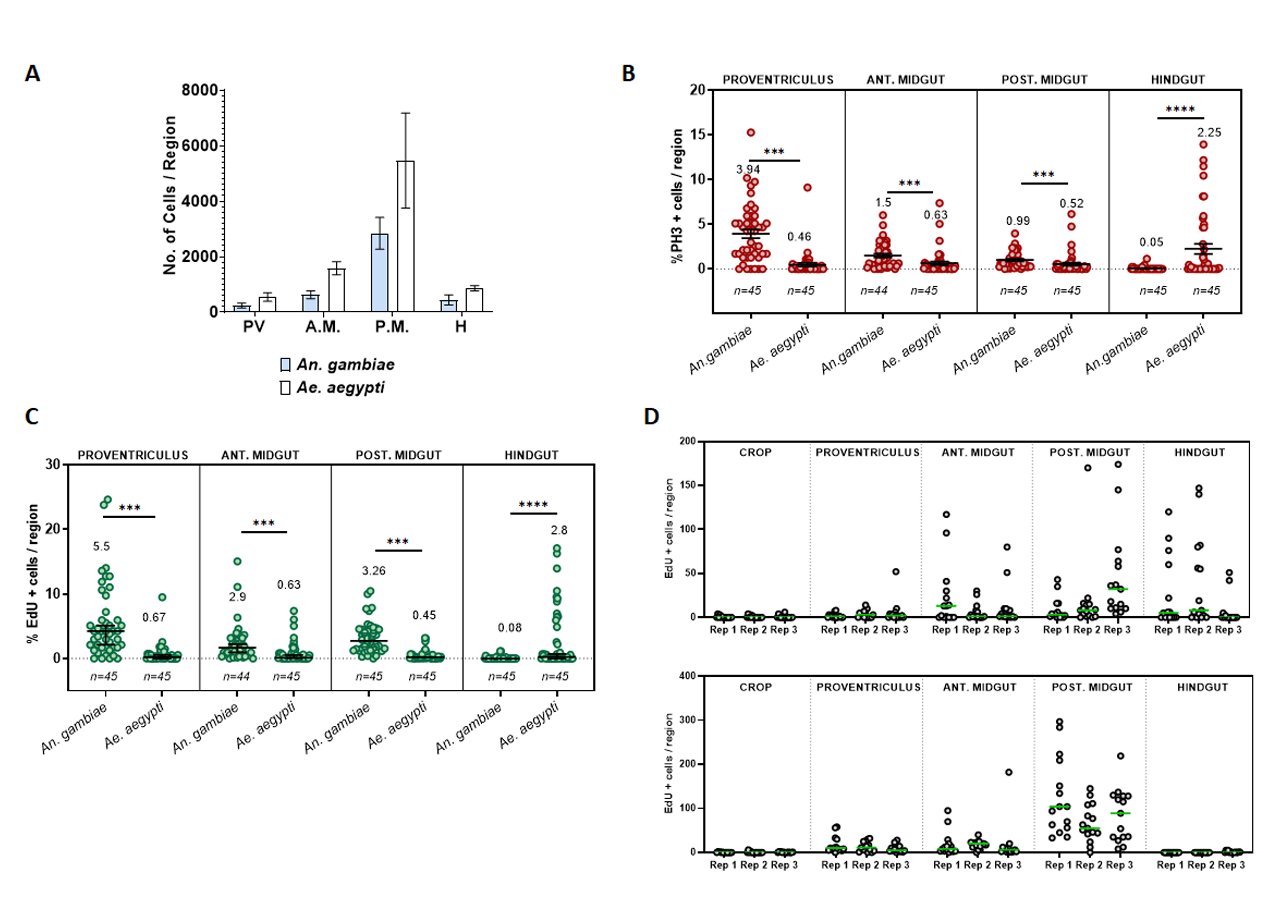

Supplement: Supplementary file 1 — Additional file 1: Supplemental Figure 1. Bacterial infection protocols. (A) Selection of an optimal dose of bacteria to use for all experiments was done by quantifying the percentage of EdU-positive cells resulting from Pseudomonas entomophila infections in Aedes aegypti using OD600 of 25, 50 and 100. An OD600 of 100 was selected due to the more consistent increase of EdU-positive cells. Results are from at least three biological replicates. Statistics: Brown-Forsythe and Welch ANOVA tests for multiple comparisons, Alpha 0.05. (B) Upper timeline shows the protocol for continuous EdU incorporation in the infection experiments: Mature, 5-day-old females were infected with a sucrose-baited solution containing P. entomophila and EdU, thereafter maintained on sucrose/EdU and dissected at 24- or 72-hours post-infection (PI). Guts were treated with a Click-iT cocktail to label EdU (green) and stained with an anti-PH3 antibody (red) and DAPI (blue). This protocol was used to infect Ae. aegypti, Aedes albopictus, Culex quinquefasciatus, Anopheles gambiae and Anopheles stephensi. In a second set of experiments (bottom timeline), used only with Ae. aegypti and An. gambiae, mosquitoes were kept on sucrose/EdU for 24 hours (including the time of infection), then switched to sucrose/BrdU. [file 12915_2023_1769_MOESM1_ESM.tif]

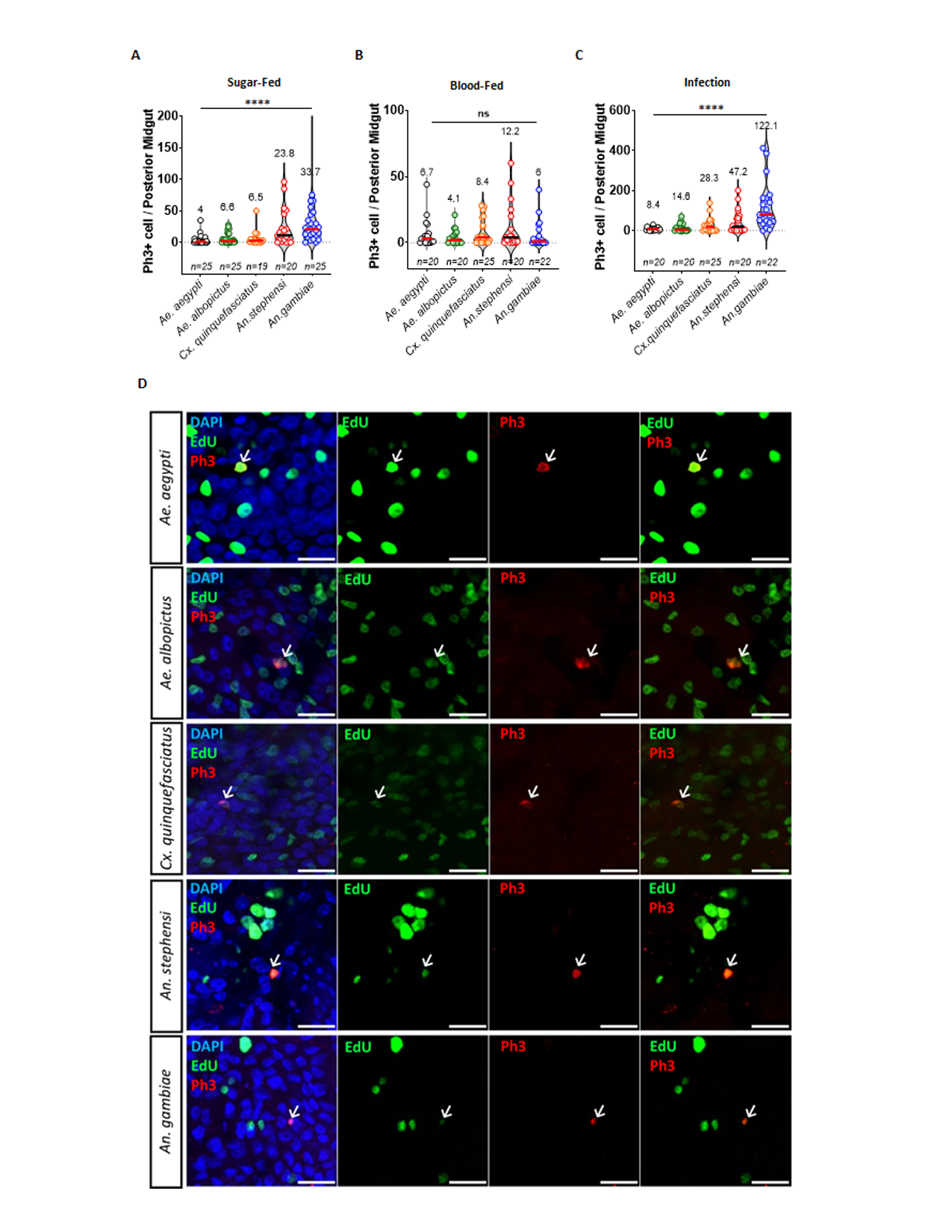

Supplement: Supplementary file 2 — Additional file 2: Supplemental Figure 2. Gating strategy for Flow Cytometry analysis. (A) All events were plotted on forward versus side scatter (FSC vs. DAPI-A) and gated to eliminate debris. This was the primary gate (H1) which included non-debris, stained events. (B) Stained events included in the primary gate (H1) were plotted on DAPI(DNA)-H vs DAPI(DNA)-A (voltage pulse area vs. height) to eliminate doublets. This secondary gate was called “non-doublets”, five thousand events were required in this gate for a sample to be included in the analysis. (C) All events in the non-doublet gate were then plotted as EdU fluorescence (y axis) vs DNA content (x axis) and gates of EdU-positive and EdU-negative events were established using samples with no EdU treatment to set up negative control values. (D) For each species, larval brains were dissected (5 per sample) and stained with DAPI for diploid controls. Overlapping histograms of the diploid control and the midgut epithelial cells of sugar-fed samples were used to identify the placement of the diploid population amongst the ones in the midgut epithelium. Cell count on the Y axis is a normalized value relative to the total cell count. Each experiment was performed in at least three biological replicates. [file 12915_2023_1769_MOESM2_ESM.tif]

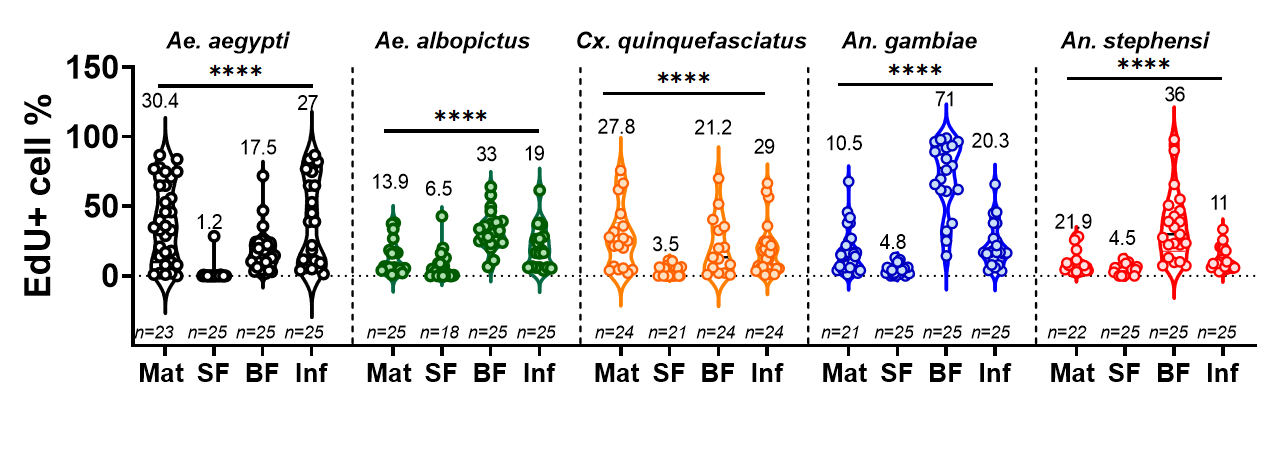

Supplement: Supplementary file 3 — Additional file 3: Supplemental Figure 3. PH3- and EdU-positive cell counts normalized to total cell number per gut region. Five-day-old females were kept on 10% sucrose with 100 µM EdU for 72 hours. Full guts were dissected on ice-cold PBS and immune-stained to label mitosis using PH3 antibody (1:500). Counts of DAPI+ nuclei were used to estimate total cell number per region (A). Aedes aegypti guts had approximately double the quantity of cells in the Anopheles gambiae guts. PH3-positive cells presented in percentage are shown in (B), and EdU-positive cells are presented in (C). Values on top indicate mean values, and error bars are SEM. Mann-Whitney tests were performed between species and P values < 0.05 were considered significantly different. Each experiment was performed in biological triplicates. (D) Individual cell counts of EdU positive cells in each biological replicate, presented by total number of cells per region in Ae. aegypti and An. gambiae, show that there was no batch effect detectable when. [file 12915_2023_1769_MOESM3_ESM.tif]

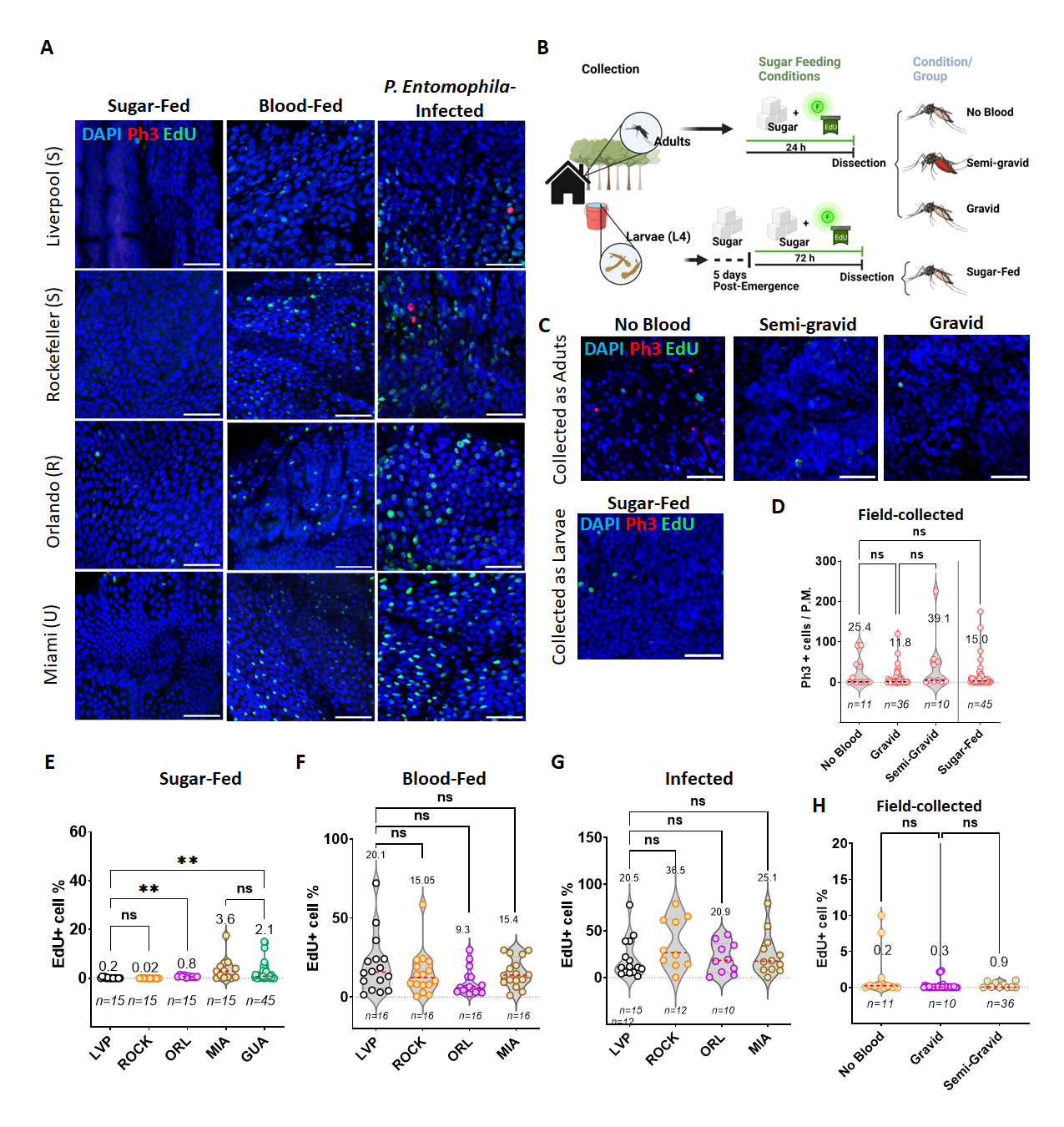

Supplement: Supplementary file 4 — Additional file 4: Supplemental Figure 4. PH3-positive cells were observed in Aedes, Culex and Anopheles mosquitoes. Fixed tissue from eight-day-old females that had either been sugar-fed, blood-fed or infected, and maintained on 10% sucrose with 100 µM EdU for 72 hours, was treated uniformly across species, through permeabilization, Click-it, blocking and antibody staining protocols. Primary antibody, rabbit anti-PH3 (1:500) and secondary antibody, goat anti-rabbit Alexa 555 (1:2000), were used for all samples. Quantification of PH3-positive cells per posterior midgut in sugar-fed (A), blood-fed (B), and infected (C) mosquitoes was done in Aedes aegypti, Aedes albopictus, Culex quinquefasciatus, Anopheles gambiae and Anopheles. stephensi. Values on top indicate mean values, and error bars are SEM. Mann-Whitney tests were performed between species and P < 0.05 was considered significantly different. Each experiment was performed in biological triplicates. Representative images of PH3- and EdU-positive cells for each species, under infection conditions, are shown in (D). (Scale bar = 50 µm) and white arrows indicate yellow (double-stained) cells. [file 12915_2023_1769_MOESM4_ESM.tif]

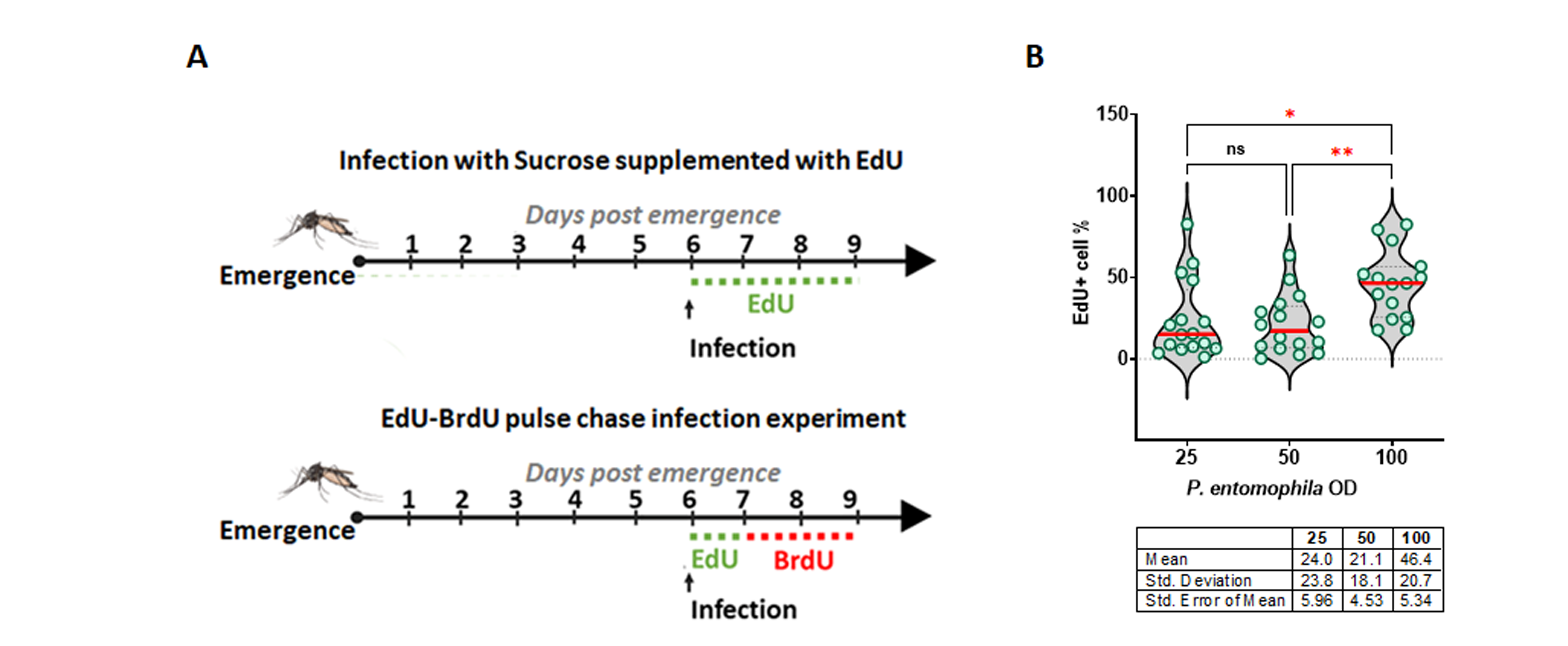

Supplement: Supplementary file 5 — Additional file 5: Supplemental Figure 5. Percentages of EdU-positive cells in Aedes, Culex and Anopheles mosquitoes changed with different physiological conditions. Fixed tissue from either three days old (maturation group, Mat) and eight-day-old females that had either been sugar-fed (SF), blood-fed (BF) or infected (Inf), and maintained on 10% sucrose with 100 µM EdU for 72 hours, was treated with a Click-it reaction to visualize cells that had incorporated EdU through the synthesis of DNA. Results are from at least three biological replicates. Values on top indicate mean values and error bars are SEM. Statistics: one-way ANOVA, P <0.001. [file 12915_2023_1769_MOESM5_ESM.tif]

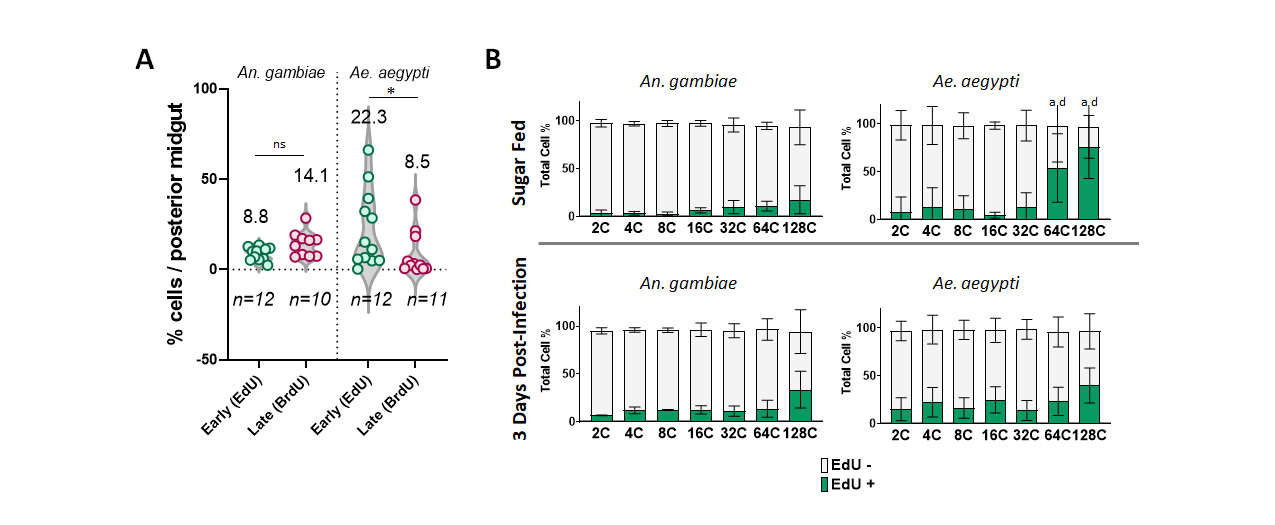

Supplement: Supplementary file 6 — Additional file 6: Supplemental Figure 6. The midgut epithelium of adult female mosquitoes shows conserved responses under sugar-fed, blood-fed and infected conditions in different strains of Aedes aegypti. (A) Five-day old female Ae. aegypti of the Liverpool, Rockefeller, Orlando and Miami strains were maintained for 72 hours on a diet of 10% sucrose supplemented with EdU for the sugar-fed group. Similarly, five-day-old females were either blood-fed on bovine blood or infected with P. entomophila and maintained on 10% sucrose with EdU for 72 hours prior to dissection. Guts were treated with a Click-iT cocktail to label EdU (green) and stained with an anti-PH3 antibody (red) and DAPI (blue), (scale bar = 50 µm). Additional Ae. aegypti were field collected in the department of Chiquimula, Guatemala in adult and larval stages (B). The adults were dissected 24 hours after collection to obtain representative images of the state of gut epithelial dynamics in the wild, and larvae were transported to the insectary at Del Valle de Guatemala University to be maintained until 5 days post-emergence. Representative images of the midgut epithelium of field-collected mosquitoes are shown in (C), including mosquitoes collected as adults (top) and larvae (bottom). Adults were classified by gut content/gravidity as “blood-fed”, “semi-gravid” or “gravid”. Larvae were reared to adulthood under laboratory conditions. Total counts of PH3-positive cells on these groups are shown in (D). Values on top indicate mean values, and error bars are SEM. Quantifications of EdU-positive cells in the region of interest of all laboratory-reared mosquitoes (including those reared from field-collected larvae) are shown in (E). Consistent with prior results, Liverpool, Rockefeller and Orlando strains showed average numbers of EdU-positive cells below 1%. Miami strain and field-collected mosquitoes from Guatemala showed significantly higher numbers. The percentages of EdU-positive cells were not signifi [file 12915_2023_1769_MOESM6_ESM.tif]

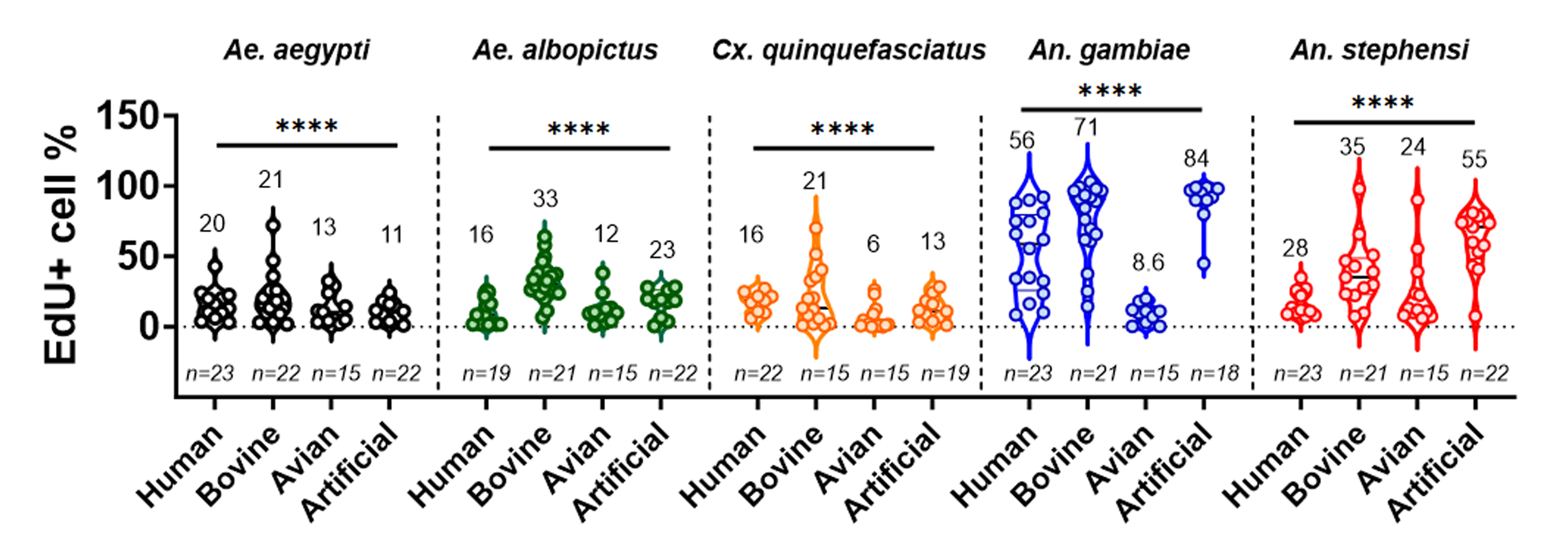

Supplement: Supplementary file 7 — Additional file 7: Supplemental Figure 7. EdU and BrdU incorporation in orally infected mosquitoes through time and after three days by ploidy. Fixed midguts from eight-day-old females that had been infected with Pseudomonas entomophila and maintained on 10% sucrose with 100 µM EdU for 24 hours and, subsequently, on 10% sucrose with 1 µM BrdU for 48 hours, were treated with a Click-iT reaction for EdU (green) and immunostaining for BrdU (red) (A). Different cell populations, based on DNA content, had incorporated EdU after 72 hours of feeding with sucrose supplemented with EdU (B). The upper row presents the percentages of EdU-positive cells per cell population in sugar-fed mosquitoes (unchallenged) and the lower row presents percentages of EdU-positive cells per cell population in P. entomophila-infected mosquitoes. Samples consisted of pools of 8 posterior midguts, n=9, from at least three biological replicates. [file 12915_2023_1769_MOESM7_ESM.tif]

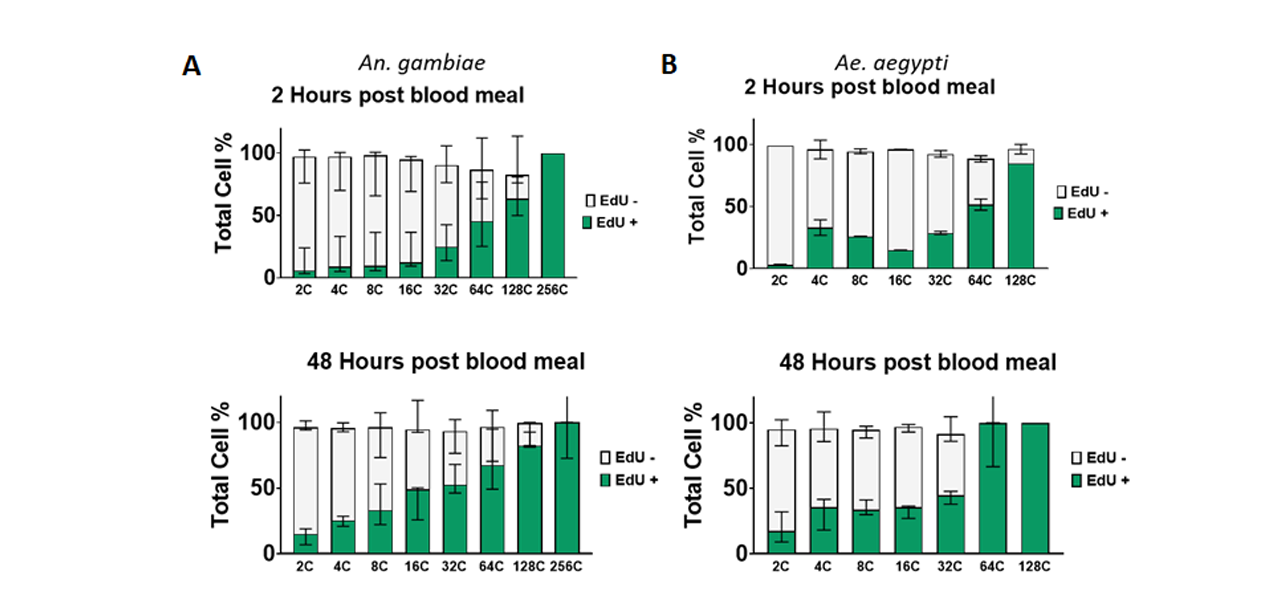

Supplement: Supplementary file 8 — Additional file 8: Supplemental Figure 8. EdU incorporation in mosquitoes blood-fed with different blood-sources. Five-day-old female mosquitoes were blood-fed on human, bovine or avian blood, as well as on the artificial diet SkitoSnack (all supplemented with 100 µM EdU) and then maintained on 10% sucrose with 100 µM EdU for 72 hours (A-E). Different blood sources resulted in different responses from the same mosquito species. Values on top indicate mean values, and error bars are SEM. Mann-Whitney tests were performed between species and P < 0.05 was considered significantly different. Each experiment was performed in biological triplicate. [file 12915_2023_1769_MOESM8_ESM.tif]

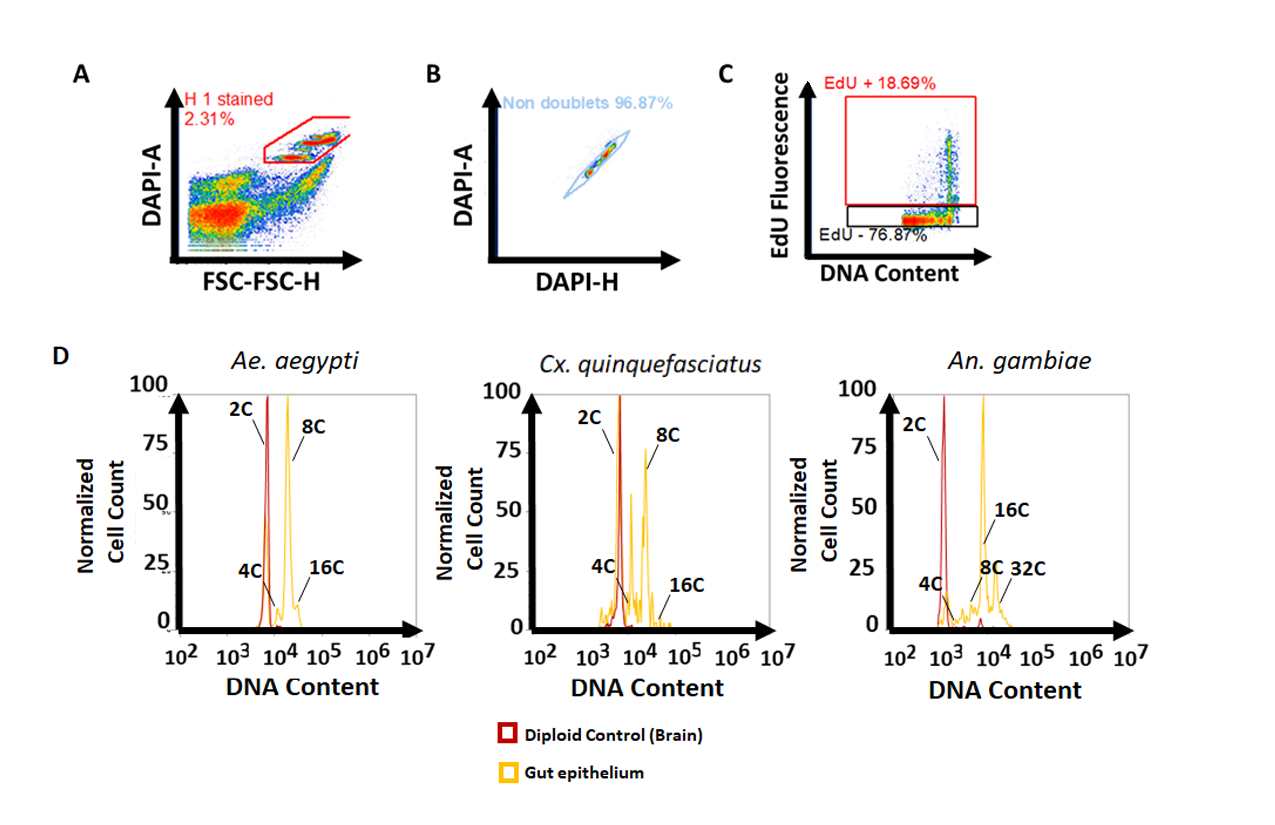

Supplement: Supplementary file 9 — Additional file 9: Supplemental Figure 9. EdU incorporation in the midgut of Anopheles gambiae and Aedes aegypti after blood feeding by ploidy. (A) In the epithelium of An. gambiae, the number of larger ploidy cells showing incorporation of EdU is high even at two hours after the blood meal. The percentage of EdU-positive cells in all cell populations is significantly higher by the end of the digestive process. (B) In Ae. aegypti, the overall quantity of EdU-positive cells is less than in An. gambiae (Fig. 6C), but there is also a significant incorporation of EdU in all the cell populations. Samples consisted of pools of 8 posterior midguts, n=9 samples per condition, from at least three biological replicates. [file 12915_2023_1769_MOESM9_ESM.tif]
